# Establishing a standardised approach for the measurement of neonatal noxious-evoked brain activity in response to an acute somatic nociceptive heel lance stimulus

**DOI:** 10.1016/j.cortex.2024.05.023

**Published:** 2024-10

**Authors:** Marianne Aspbury, Roshni C. Mansfield, Luke Baxter, Aomesh Bhatt, Maria M. Cobo, Sean P. Fitzgibbon, Caroline Hartley, Annalisa Hauck, Simon Marchant, Vaneesha Monk, Kirubin Pillay, Ravi Poorun, Marianne van der Vaart, Rebeccah Slater

**Affiliations:** a Department of Paediatrics, University of Oxford, Oxford, UK; b Newborn Care Unit, John Radcliffe Hospital, Oxford University Hospitals NHS Foundation Trust, Oxford, UK; c Universidad San Francisco de Quito USFQ, Colegio de Ciencias Biologicas y Ambientales, Quito, Ecuador; d Wellcome Centre for Integrative Neuroimaging, FMRIB, Nuffield Department of Clinical Neurosciences, University of Oxford, UK; e Children's Services, Royal Devon & Exeter NHS Foundation Trust, Exeter, UK; f College of Medicine & Health, University of Exeter, Exeter, UK

**Keywords:** ERP, Evoked potential, Reliability, Biomarker, Nociception

## Abstract

**Background:**

Electroencephalography (EEG) can be used in neonates to measure brain activity changes that are evoked by noxious events, such as clinically required immunisations, cannulation and heel lancing for blood tests. EEG provides an alternative approach to infer pain experience in infants compared with more commonly used behavioural and physiological pain assessments. Establishing the generalisability and construct validity of these measures will help corroborate the use of brain-derived outcomes to evaluate the efficacy of new or existing pharmacological and non-pharmacological methods to treat neonatal pain. This study aimed to test whether a measure of noxious-evoked EEG activity called the noxious neurodynamic response function (n-NRF), that was originally derived in a sample of term-aged infants at the Oxford John Radcliffe Hospital, UK, in 2017, can reliably distinguish noxious from non-noxious events in two independent datasets collected at University College London Hospital and at Royal Devon & Exeter Hospital. We aimed to reproduce three published results that use this measure to quantify noxious-evoked changes in brain activity. We used the n-NRF to quantify noxious-evoked brain activity to test (i) whether significantly larger noxious-evoked activity is recorded in response to a clinical heel lance compared to a non-noxious control heel lance procedure; (ii) whether the magnitude of the activity evoked by a noxious heel lance is equivalent in independent cohorts of infants; and (iii) whether the magnitude of the noxious-evoked brain activity increases with postmenstrual age (PMA) in premature infants up to 37 weeks PMA. Positive replication of these studies will build confidence in the use of the n-NRF as a valid and reliable pain-related outcome which could be used to evaluate analgesic efficacy in neonates. The protocol for this study was published following peer review (https://doi.org/10.17605/OSF.IO/ZY9MS).

**Results:**

The n-NRF magnitude to a noxious heel lance stimulus was significantly greater than to a non-noxious control heel lance stimulus in both the UCL dataset (*n* = 60; mean difference .88; 95% confidence interval (CI) .64–1.13; *p* < .0001) and the Exeter dataset (*n* = 31; mean difference .31; 95% CI .02–.61; *p* = .02). The mean magnitude and 90% bootstrap confidence interval of the n-NRF evoked by the heel lance did not meet our pre-defined equivalence bounds of 1.0 ± .2 in either the UCL dataset (*n* = 72; mean magnitude 1.33; 90% bootstrapped CI 1.18–1.52) or the Exeter dataset (*n* = 35; mean magnitude .92, 90% bootstrapped CI .74–1.22). The magnitude of the n-NRF to the noxious stimulus was significantly positively correlated with PMA in infants up to 37 weeks PMA (*n* = 65; one-sided Pearson's R, adjusted for site: .24; 95% CI .06–1.00; *p* = .03).

**Conclusions:**

We have reproduced in independent datasets the findings that the n-NRF response to a noxious stimulus is significantly greater than to a non-noxious stimulus, and that the noxious-evoked EEG response increases with PMA. The pre-defined equivalence bounds for the mean magnitude of the EEG response were not met, though this might be due to either inter-site differences such as the lack of calibration of devices between sites (a true negative) or underpowering (a false negative). This reproducibility study provides robust evidence that supports the use of the n-NRF as an objective outcome for clinical trials assessing acute nociception in neonates. Use of the n-NRF in this way has the potential to transform the way analgesic efficacy studies are performed.

## 1 Introduction

### 1.1 Background and rationale

Reproducibility studies can establish whether clinical research findings are generalisable (Goodman, Fanelli, & Ioannidis, 2016; Nosek & Errington, 2020). Positive replication builds confidence in conclusions and, conversely, negative replication can prevent potential harms and avoid time-wasting caused by pursuing false findings. When conducting reproducibility studies, pre-registering the study design and planned analytical approaches builds trust in reported results and helps avoid subconscious or conscious bias, and changing research methods to fit desired outcomes (Bishop, 2019).

The study of neonatal pain will benefit from reproducibility studies. Currently pain management presents a major challenge in neonatal care and analgesic provision is infrequently and inconsistently provided during essential medical interventions (Carbajal et al., 2008; Flint et al., 2018). In part, this is due to difficulties in measuring pain and assessing the efficacy of analgesics in the non-verbal neonatal population (Slater, Moultrie, Bax, van den Anker, & Bhatt, 2020). While numerous clinical tools have been developed to calculate pain scores in neonates (Carter & Brunkhorst, 2017; Melo, Lélis, Moura, Cardoso, & Silva, 2014), these measures are often reliant on behavioural and physiological observations that may not be sensitive or specific enough to (i) distinguish pain from distress (Ranger, Johnston, & Anand, 2007), or (ii) to be primary outcome measures in clinical trials that aim to objectively assess the efficacy of analgesic interventions (Slater et al., 2020). An alternative approach has been to consider changes in brain activity evoked by noxious clinical procedures (Hartley et al., 2018; Slater, Cornelissen, et al., 2010).

Over the last 15 years, brain-derived approaches have been used to quantify how the infant brain responds to acute noxious medical procedures, such as heel lancing and inoculations (Slater, Worley, et al., 2010; Verriotis et al., 2015). These brain-derived approaches have been used as the primary outcome measure to test the efficacy of analgesics and pain management interventions in clinical trials (Hartley et al., 2018; Hauck et al., 2024; Slater, Cornelissen, et al., 2010). Current and ongoing discussions with the FDA Division of Pediatrics and Maternal Health (DPMH) in collaboration with the Division of Anesthesiology, Addiction Medicine and Pain Medicine (DAAP) (https://www.fda.gov/drugs/news-events-human-drugs/fda-m-cersi-analgesic-clinical-trial-designs-extrapolation-and-endpoints-patients-birth-less-two) are focussed on the use of brain-derived metrics in infants to improve analgesic drug development, with a specific focus on analgesic medications with known mechanisms of action such as NSAIDs, acetaminophen, local anaesthetics and opioids. It is therefore imperative that proxy pain measures that rely on brain-derived metrics are valid, reliable, and appropriate for use in large-scale clinical trials if they are to be a standard by which the efficacy of new or existing drugs can be evaluated in the neonatal population.

An EEG-derived pattern of noxious-evoked activity has been observed in multiple settings, including in the UK at University College London Hospital (Fabrizi et al., 2011; Jones et al., 2017; Slater, Worley, et al., 2010) and the John Radcliffe Hospital, Oxford (Gursul et al., 2018; Hartley et al., 2016, 2017), in Israel at Soroka Medical Centre in Beer Sheva (Maimon et al., 2013), in Switzerland at the University Basel Hospital (Kasser et al., 2019) and in the USA at the Nationwide Children's Hospital (Maitre et al., 2017) – and is the most commonly used neuroimaging approach to study pain in infants (Benoit, Martin-Misener, Newman, Latimer, & Campbell-Yeo, 2017). Across multiple settings, a range of biological factors have been identified that modulate noxious-evoked brain activity, including premature birth (Fabrizi et al., 2011; Green et al., 2019; Hartley et al., 2016; Schmidt Mellado et al., 2022), mode of delivery (Kasser et al., 2019), stress (Jones et al., 2017), inflammation (Cobo, Green, et al., 2022) and sex (Verriotis et al., 2018). However, noxious-evoked EEG activity can be quantified in various ways, which hampers the comparison of effect sizes across different studies. To quantify and standardise changes in brain activity that arise following noxious clinical procedures, an EEG-based “template” measure that quantifies the magnitude of the noxious-evoked activity at a single central electrode site has been developed (Hartley et al., 2017). We term this “template” the noxious neurodynamic response function (n-NRF),^3^ and this measure constitutes a waveform which can be scaled to fit evoked activity in an EEG recording. We have previously demonstrated that the n-NRF magnitude is (i) larger following noxious stimuli compared to arousing non-noxious sensory events (Hartley et al., 2017), (ii) is sensitive to modulation by pain management strategies (Cobo et al., 2021; Gursul et al., 2018; Hartley et al., 2017) and (iii) is moderately correlated with pain-relevant behaviour (Hartley et al., 2017). However, we have not previously investigated the consistency of these observations at different research sites. Here we have pre-registered a report which describes a multicentre study to explore the reproducibility and generalisability of the noxious-evoked EEG activity following a heel lance and to establish if reported biological observations that use this approach are generalisable across different centres.

The n-NRF was derived and validated in term and preterm infants aged 34.0–43.0 weeks' postmenstrual age (PMA) and is scaled to give an average magnitude of 1.0 in response to a heel lance performed in newborn term-aged infants (Hartley et al., 2017). This pre-registered reproducibility study had two aims. Firstly, we wanted to establish cross-centre generalisability by identifying whether the n-NRF can be reliably projected onto independent datasets. COSMIN (Consensus-based Standards for the selection of health Measurement Instruments) outlined a set of properties which should be upheld by medical instrument measurements. We used these guidelines as a basis to create testable hypotheses for two key criteria outlined by COSMIN: construct validity and reliability (Mokkink et al., 2010). To this end, we planned to use retrospective and prospective datasets collected at two different sites to (i) identify whether the magnitude of the n-NRF is significantly larger in response to a noxious compared to an innocuous procedure, which is an assessment of the construct validity of the n-NRF, and (ii) identify whether the n-NRF magnitude to a noxious procedure is consistent across sites, which is an assessment of inter-site reliability. This multi-level testing was designed to form a robust construct validity and inter-site reliability assessment, and therefore a generalisability assessment, of the n-NRF to discriminate between a noxious and an innocuous procedure in infants 34.0–43.0 weeks PMA. Secondly, there is value in exploring whether previous research findings relating to biological factors that are reported to modulate the magnitude of the EEG noxious event-related potentials are reproducible (Hartley et al., 2016; Schmidt Mellado et al., 2022). Hence, we have tested whether the magnitude of noxious-evoked brain activity following a heel lance, as quantified by the n-NRF, increases with PMA during the period of prematurity up to 37 weeks PMA in infants born at less than 36 weeks’ gestational age (GA), as reported by Schmidt Mellado and colleagues in 2022 (Schmidt Mellado et al., 2022). The study populations, samples size planning, measurement, EEG processing and pre-specified analytical approaches are described in detail in the Methods, and the precise hypotheses are stated below. It is important to note that while the hypothesis tests assess the reproducibility of a measure of noxious-evoked neonatal brain activity following a heel lance, this work does not provide evidence that these activity patterns are a direct measure of neonatal pain. In the absence of language, we cannot know whether another person is in pain and therefore the brain-derived activity characterised here reflects only a pattern of brain activity that is known to be evoked by a noxious stimulus.

To plan this study and to conduct appropriate sample size planning analyses, we initially tested the generalisability of the n-NRF in a large Oxford dataset that was independent of the data included in the original derivation of the n-NRF (Hartley et al., 2017). Results from these initial analyses are reported in Supplementary Appendix A 4 and were used for sample size planning for the subsequent studies. In this study, our proposed hypotheses were tested in two datasets collected at independent sites: (1) the “UCL dataset” is retrospective data collected and published by an independent research group at University College London Hospital (Jones et al., 2018), and (2) the “Exeter dataset” was collected at The Royal Devon & Exeter Hospital, and was planned to be collected prospectively after in-principle acceptance of our pre-registered report. The pre-registered protocol for this study was published following peer review (https://doi.org/10.17605/OSF.IO/ZY9MS).

### 1.2 Hypotheses

#### 1.2.1 Hypothesis 1

The magnitude of noxious-evoked brain activity measured using the n-NRF should be greater following a noxious heel lance compared with a non-noxious control heel lance procedure in infants 34.0–43.0 weeks PMA (described in Section 2.3). Thus, we hypothesised a significantly larger evoked n-NRF magnitude in response to the heel lance relative to the non-noxious control heel lance in infants 34.0–43.0 weeks PMA, assessed using a one-tailed paired *t*-test.

#### 1.2.2 Hypothesis 2

The group average magnitude of the noxious-evoked brain activity measured with the n-NRF should have a magnitude of 1.0 in response to a heel lance in newborn term-aged infants. Thus, we hypothesised that the average evoked n-NRF magnitude in response to the heel lance is equivalent to 1.0, assessed using the magnitude, confidence interval and pre-defined equivalence bounds.

#### 1.2.3 Hypothesis 3

The magnitude of the noxious-evoked brain activity measured with an EEG template approach in response to a heel lance has been reported to increase in premature infants with PMA up to 37.0 weeks. Thus, we hypothesised a statistically significant positive correlation between PMA and the n-NRF magnitude in response to the heel lance, assessed using a Pearson correlation test.

### 1.3 Glossary of measurement property terminology

A glossary of measurement property terminology used throughout this manuscript is provided below.

**Table:** 

Term	Definition
Validity	**Validity** is the degree to which a measurement truly represents the construct it is meant to represent (Mokkink et al., 2010). **Construct validity** is a type of validity that describes the degree to which the measurement scores confirm hypotheses that are founded on the assumption that the measurement represents a valid measure of the construct being assessed (Mokkink et al., 2010). **Known-groups validity** is the ability of the measurement method to distinguish between groups that differ in the construct being measured (Mokkink et al., 2010).
Reliability	The **reliability** of a measurement is the amount of variance between measurements that is explained by true differences between patients (Mokkink et al., 2010). Here, we were interested in **inter-site reliability** of the n-NRF.
Generalisability	The **generalisability** of a study (also known as external validity or applicability) is the extent to which the findings of a study can be correctly applied to other settings (Altman, 2001).

## 2 Methods

We report how we determined our sample size, all data exclusions, all inclusion/exclusion criteria, whether inclusion/exclusion criteria were established prior to data analysis, all manipulations, and all measures in the study.

### 2.1 Study design

For Hypothesis 1, the study uses a within-participant cross-sectional design, comparing outcomes of an observed clinical procedure to an experimental control procedure in the same participants. For Hypotheses 2 and 3, the studies use a between-participant cross-sectional design, measuring outcomes of an observed clinical procedure across participants who are each studied only once. For the UCL dataset, there were no additional interventions for any participants. All infants received standard clinical care throughout the study in accordance with local practice unit guidelines.

We had planned to start prospective data collection for the Exeter dataset following in-principle acceptance of our registered report, and to complete data collection within one year. However, following delays in the protocol registration process (protocol submitted on 25/01/2022; protocol accepted on 11/09/2022), we were unable to prospectively collect data from the protocol publication date in a timely manner. Therefore, a pragmatic decision was made to include all eligible babies who had been recruited and studied at the Exeter site since 04/06/2021.^5^ Some of these infants were enrolled in the Parental Touch Trial (Petal), a randomised controlled trial aiming to assess the effect of parental touch before a painful procedure (Cobo, Moultrie, et al., 2022). Heel lance-evoked brain activity was assessed in infants born at ≥35 weeks GA who needed a blood test within one week of birth. The stimuli and EEG recording set-up for the Petal Trial were the same as for the planned prospective study in Exeter. However, infants in the Petal Trial were randomised 1:1 to receive parental touch either before (intervention group) or after (control group) the heel lance (Cobo, Moultrie, et al., 2022).

### 2.2 Participants

**Fig. 1:**
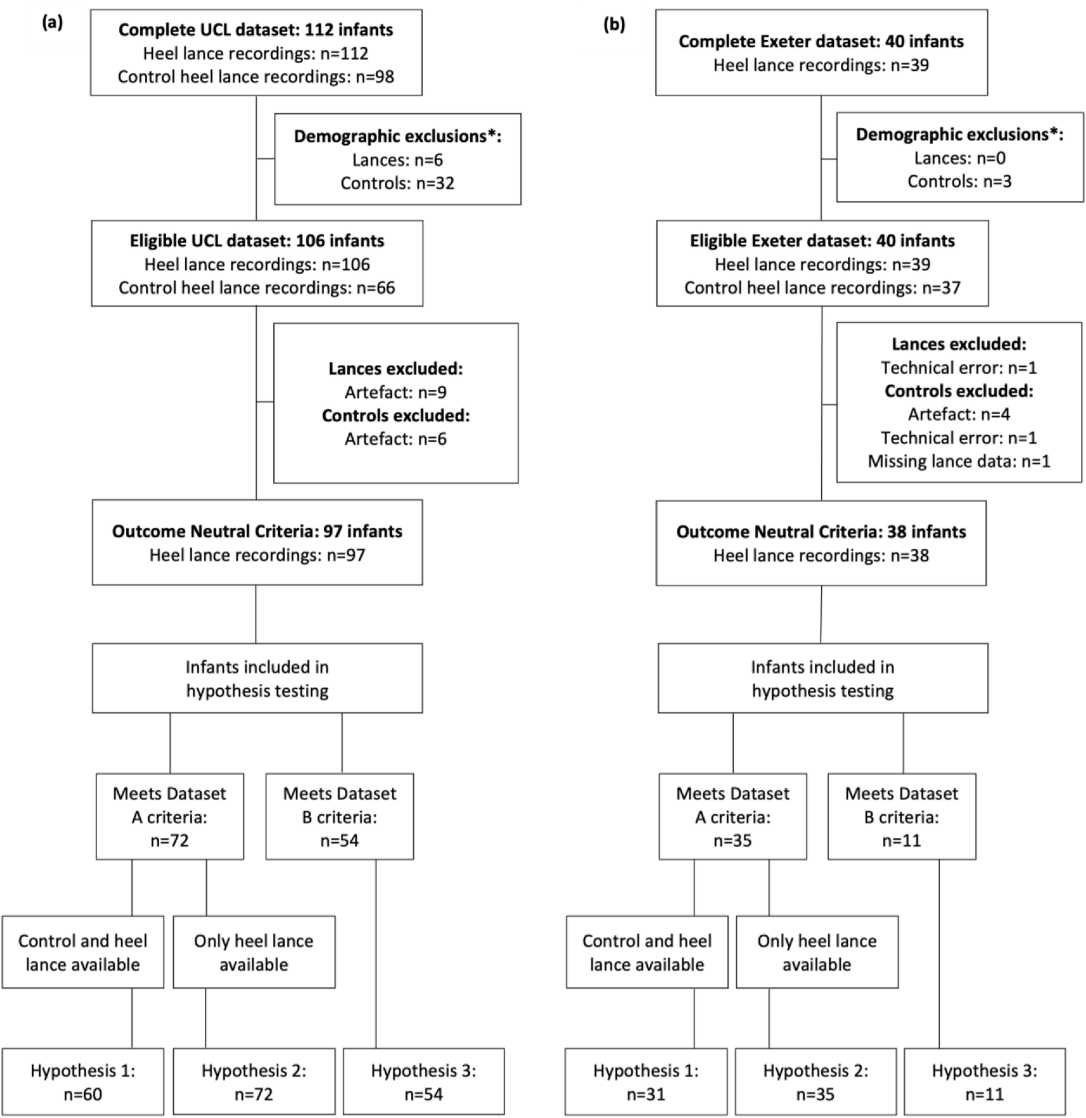
Number of infants in each dataset: UCL **(a)** and Exeter **(b)**. ∗Recordings which do not fit demographic inclusion criteria for any hypothesis. Only control heel lance data eligible for inclusion in Hypothesis 1 analyses were included in Outcome Neutral Criteria testing.

The same data inclusion and exclusion criteria were applied for each dataset, although inclusion criteria depended on the hypothesis in question. Both Hypotheses 1 and 2 shared the same demographic inclusion criteria, however the third hypothesis included a different sample. Inclusion and exclusion criteria closely match key variables in the prior studies that we are reproducing, such as the age range of the infants. We conducted our analyses in different subsamples of the UCL Dataset (UCL Dataset A and Dataset B) and Exeter Dataset (Exeter Dataset A and Dataset B) to satisfy the inclusion criteria for each hypothesis. An overview of the planned and included data subsamples can be found in Table 1 and Fig. 1, respectively.

**Table 1:** Summary of data from UCL and Exeter required for each stage of analysis. The number of infants required at each stage of analysis is indicated in the final column, and these numbers were planned to be inflated by 10% for a prospective study, to account for data loss, artefact or participant withdrawal, as explained in Section 2.4. All infant data in Datasets A met demographic inclusion criteria for Hypothesis 1 and Hypothesis 2. For Hypothesis 1, only the infants in Datasets A that had a control lance and a heel lance that met EEG quality criteria were included (UCL *n* = 60; Exeter *n* = 31), whereas for Hypothesis 2, any eligible infants in Datasets A with heel lance data only were included (UCL *n* = 72; Exeter *n* = 35). For Hypothesis 3, all infant data in Datasets B were used.

Analysis stage	Inclusion criteria	EEG data required	UCL or Exeter datasets	Sample size estimation
Outcome Neutral Criteria	Any data eligible for any hypothesis test	Heel lance and control heel lance data analysed separately	UCL and Exeter Datasets A and B analysed together	Not applicable
Hypothesis 1	Birth GA: 31.0–43.0 weeks Study PMA: 34.0–43.0 weeks	Heel lance and control heel lance data	UCL and Exeter Dataset A analysed separately	*N* = 35 (+10%)
Hypothesis 2	Birth GA: 31.0–43.0 weeks Study PMA: 34.0–43.0 weeks	Heel lance data only	UCL and Exeter Dataset A analysed separately	*N* = 73 (+10%)
Hypothesis 3	Birth GA: <36.0 weeks Study PMA: ≤37.0 weeks	Heel lance data only	UCL and Exeter Dataset B analysed together	*N* = 38 (+10%)

#### 2.2.1 Participant datasets

##### 2.2.1.1 The UCL dataset

The UCL dataset is available via ReShare. Users require authorised access due to the sensitivity of the data (Jones et al., 2018, UK Data Service https://doi.org/10.5255/UKDA-SN-853204). We included all available data from this retrospective dataset to test each hypothesis.

###### 2.2.1.1.1 UCL dataset A. Data inclusion for Hypotheses 1 and 2

For both these hypotheses we included healthy infants who were 31.0–43.0 weeks GA at birth and were 34.0–43.0 weeks PMA at time of study, without restriction on postnatal age (PNA). This reflects the demographics in the original Hartley validation study (Hartley et al., 2017), as well as the pilot data used to power these hypotheses (Supplementary Appendix A, A1.1). For Hypothesis 1, infants needed to have both a control heel lance and a heel lance procedure recorded with EEG that passed the rejection criteria (Section 2.6) to be included. For Hypothesis 2, only a heel lance procedure meeting these requirements was necessary.^6^

###### 2.2.1.1.2 UCL dataset B. Data inclusion for Hypothesis 3

We included infants who were born at less than 36.0 weeks GA and studied up to and including 37.0 weeks PMA, without restriction on PNA. This age range reflects those studied by Schmidt Mellado, who found that noxious-evoked response following a heel lance correlates with PMA at study in premature infants in this range (Schmidt Mellado et al., 2022). Infants included for Hypothesis 3 only required an EEG recording during a heel lance procedure which passed EEG rejection criteria; data quality of the control heel lance was therefore not assessed for inclusion.

##### 2.2.1.2 The Exeter Dataset

Ethical approval for the Exeter study was granted by the NHS Research Ethics Committee as part of an ongoing study (reference 12/SC/0447). Written parental consent was obtained from parents prior to each study and studies conformed with the declaration of Helsinki. Pilot data collection began on 04/06/2021 for training purposes, to ensure adequate familiarity with EEG recording equipment and protocol in advance of the planned prospective study. As stated in Section 2.1, data collection for the planned analysis was not intended to begin until in-principle acceptance of the report, but due to a combination of delays in in-principle acceptance and restrictions on time and resources, we have included all babies studied in Exeter since 04/06/2021. Therefore, as stated, in Section 2.1, we have pragmatically included infants recruited as part of the Petal Trial (NHS Research Ethics Committee reference 21/LO/0523).

Ethical conditions limit the sharing of the raw Exeter data publicly. Data requests should be made to the senior author (rebeccah.slater@paediatrics.ox.ac.uk). Data will be shared with investigators whose proposed use of the data has been approved by the senior author Professor Rebeccah Slater, to achieve aims in the approved proposal.

###### 2.2.1.2.1 Exeter Dataset A. Data inclusion for Hypothesis 1

In Exeter, we aimed to recruit and study a minimum required sample of *n* = 40 infants to test Hypothesis 1 (Section 2.4.1).

We included infants who were 31.0–43.0 weeks GA at birth and were 34.0–43.0 weeks PMA at time of study, without restriction on PNA. This reflects the demographics supported in the original Hartley study (Hartley et al., 2017), as well as the sample of pilot data used to power this hypothesis (Supplementary Appendix A, Section A1.1). For hypothesis 1, infants needed to have both a control heel lance and a heel lance procedure recorded with EEG that passed the rejection criteria (Section 2.6) to be included. For Hypothesis 2, only a heel lance procedure meeting these requirements was necessary.

###### 2.2.1.2.2 Exeter Dataset B. Data inclusion for Hypothesis 3

We did not plan to recruit further infants to test this hypothesis, as we planned to include all relevant infants from our recruited sample of 40 infants in Exeter whose demographics are described above. For this hypothesis, we included infants born prematurely at a GA less than 36.0 weeks and with PMA at study of up to and including 37.0 weeks, to reflect the hypothesis that the noxious-evoked response following a heel lance correlates positively with age at study in premature infants in this range, which would replicate our previous finding (Schmidt Mellado et al., 2022).

These infants only needed an EEG recording during a heel lance procedure which passed EEG rejection criteria; therefore, data quality of control heel lance was not assessed for inclusion. The majority of data to test Hypothesis 3 was sourced from the retrospective UCL dataset, and we planned to include any additional data from the prospective Exeter dataset to maximise the available data. Data from UCL alone was predicted to at least meet the calculated sample size requirement of *n* = 38 infants (Section 2.4.3).

#### 2.2.2 Data exclusion criteria

Infants were excluded if they had any known or suspected neurological condition such as (but not limited to) hypoxic ischemic encephalopathy (HIE) or intraventricular haemorrhage (IVH) of grade 2 or higher, if they received analgesics in the 24 h preceding the study, if there was known maternal substance abuse, or if they were a repeat study of an infant who was already included. Clinical data with some information about these diagnoses were available in the UCL dataset. However, if information was limited, then infant EEG data were preferentially retained for analysis to preserve sample size.^7^ We planned to exclude infants if they received an intervention other than standard clinical care which was being assessed for efficacy in reducing their noxious-evoked response, but as stated in Section 2.1, we included all babies involved in the Petal Trial, regardless of trial arm, since no difference was found in noxious-evoked EEG response to a heel lance regardless of when parents stroked their babies (Hauck et al., 2024).

EEG traces for the remaining infants were then pre-processed and analysed, following EEG rejection procedures to remove artefactual epochs as outlined in Section 2.6. Furthermore, infants were excluded after pre-processing if they did not have required EEG recordings after rejection (a heel lance and control heel lance for hypotheses 1; only a heel lance for Hypothesis 2 and 3), or if there were any other technical problems with the data for that infant (e.g., missing required data, unable to access recordings due to data corruption).

#### 2.2.3 Data replacement

We did not do any data replacement during recruitment in Exeter; we planned to recruit 10% more infants than the calculated sample sizes to account for data artefacts and participant withdrawal. Data replacement is not relevant for the UCL dataset, as we did not add to this retrospective dataset.

### 2.3 Noxious and innocuous stimuli

#### 2.3.1 Heel lance

The noxious stimulus used throughout is the heel lance, which is a clinically required procedure that involves puncturing the skin on the heel in order to obtain blood for analysis, and which is one of the most common invasive procedures performed in the NICU (Courtois et al., 2016). In all cases, the heel lances were carried out due to clinical need, and not solely for research purposes.

#### 2.3.2 Control heel lance

As a control to the noxious stimulus, the control heel lance was used, which is designed to replicate the non-noxious aspects of the heel lance procedure. The control heel lance was performed in the same conditions as the heel lance, however the control involves rotating the heel lance by 90°, so that the blade is released into the air and not the infant's foot. This removes the noxious skin-break whilst maintaining the auditory and tactile stimulation of the procedure.

### 2.4 Power analyses and sample size planning

Having demonstrated consistency of the original publication findings (Hartley et al., 2017; Schmidt Mellado et al., 2022) in our pilot data using a large locally-acquired independent Oxford dataset (Supplementary Appendix A, A1.1), we used this independent data to estimate effect sizes for the sample size planning for testing each hypothesis (Supplementary Appendix A, A1.3). These power calculations are for the relevant statistical analyses to test each hypothesis, as described in Section 2.8.

#### 2.4.1 Sample size planning for testing Hypothesis 1

To test whether there is a significant difference between heel lance and control heel lance response in our within-participant design, we calculated our required sample size with the software G∗Power for a one-tailed *t*-test of matched pairs (Faul, Erdfelder, Buchner, & Lang, 2009). Using the effect size of .589 calculated from the pilot data (Supplementary Appendix A, A1.3.3), a power of 90%, and a reduced alpha rate of .02 to account for positive bias in publication, we need 35 infants to test this hypothesis. To compensate for data loss due to artefact and participant withdrawal, the planned sample size was inflated by 10%, resulting in a total sample size of 40 infants which would be required for a prospective study.

#### 2.4.2 Sample size planning for testing Hypothesis 2

To test the equivalence of the n-NRF magnitude in response to a heel lance to 1.0, we calculated the mean magnitude and 90% confidence interval of the heel lance response in each independent dataset and determined whether this sits within the equivalence bounds of .8–1.2. According to FDA guidelines (Center for Drug Evaluation and Research, FDA, 2001; Davit et al., 2009) for planning bioequivalence studies, two measures which are within ±20% of each other can be considered equivalent. In tests of equivalence, one can consider whether the 90% confidence interval (CI) around the result is within the acceptable boundary, which in this case is a ±20% difference of 1.0 (Center for Drug Evaluation and Research, FDA, 2001; Davit et al., 2009; Schuirmann, 1987). Therefore, if the 90% CI for a group mean n-NRF magnitude in response to a heel lance sits within .80–1.20 then this group mean value can be considered equivalent to a value of 1.0.

To plan the sample size for this hypothesis testing, we pragmatically used Accuracy in Parameter Estimation (AIPE) analysis, which allows one to calculate the sample size that should result in a specified confidence interval width for a mean result (Kelley, 2007a; 2007b). In this case, we plan for a 90% confidence interval width no greater than .4; i.e., ±20% around 1.0, to confirm our hypothesis. The inputs of AIPE sample size planning are the population mean and standard deviation of the heel lance response in the pilot data (Supplementary Appendix A, A1.3.4), the desired accuracy width (.4), and confidence interval level (90%). We have calculated the required sample size from AIPE using the MBESS library in R (Kelley, 2007b, 2021) which suggests that we need to include 73 infants for a 90% CI of mean heel lance response with an accuracy width of .4. To compensate for data loss due to artefact and participant withdrawal, the sample size was inflated by 10%, resulting in a total sample size of 80 infants which would be required for a prospective study.

#### 2.4.3 Sample size planning for testing Hypothesis 3

To calculate the required sample size for a one-tailed Pearson correlation test, we used G∗Power for a calculation under the Exact test family and Correlation: Bivariate normal model statistical test (Faul et al., 2009). Using the correlation between PMA and heel lance response in the pilot data, Pearson *R* = .51 (Supplementary Appendix A, A1.3.5), a power of 90% and alpha = .02, with a null result represented by Pearson *R* = 0, the required sample size to test this hypothesis is 38 infants. To compensate for data loss due to artefact and participant withdrawal, the sample size was inflated by 10%, resulting in a total sample size of 42 infants which would be required for a prospective study.

### 2.5 Study protocol for data collection (Exeter Dataset)

#### 2.5.1 Participants

Individual infants recruited in Exeter may have been studied on more than one occasion, however only data from the first useable test occasion was included in this study. Infants received standard clinical care throughout the procedures in accordance with local practice guidelines. However, as stated in Section 2.1, some infants (29 of the 40 infants recruited in Exeter) were enrolled in the Petal Trial, and 15 received parental stroking as an intervention prior to the heel lance, while 14 received parental stroking as a control after the heel lance (Hauck et al., 2024). Full participant inclusion and exclusion criteria are presented in Section 2.2.

#### 2.5.2 Noxious and innocuous stimuli

Participants received a heel lance and control heel lance stimulus to the foot, as described in Section 2.3. Heel lances were only performed if clinically required and were carried out by a clinically-trained investigator. No extra heel lances for blood tests or other noxious stimuli were performed solely for research purposes. If the clinician performed multiple heel lances to collect enough blood, data was included from only the first heel lance where data were useable.

#### 2.5.3 EEG setup

The heel lance and control heel lance stimuli were linked electronically to the EEG recording equipment to precisely time each stimulus and mark the concurrent EEG at the time of each stimulus. This improves accuracy and precision of the marked stimuli, reduces human error, and avoids disrupting clinical practice. This time-locking method has been used and described in previous studies (Slater, Cornelissen, et al., 2010; Slater, Worley, et al., 2010; Worley, Fabrizi, Boyd, & Slater, 2012) and was also used and described by researchers for the UCL data collection (Jones et al., 2018). EEG recording equipment remained connected throughout the clinical procedure to avoid disruption to the infant's state.

Electrophysiological activity was acquired with a Compumedics Grael V2 EEG System with a bandwidth from DC - 400 Hz and a sampling rate of 2048 Hz. CURRYscan8 neuroimaging suite (Compumedics Neuroscan) was used to record the activity. All equipment conformed to the electrical safety standard for medical devices, IEC 60601-1. Eight EEG recording electrodes were positioned on the scalp at Cz, CPz, C3, C4, FCz, T3, T4 and Oz according to the modified international 10–20 System. Reference and ground electrodes were placed at Fz and the forehead respectively. EEG conductive paste was used to optimise contact with the scalp. All impedances were reduced to approximately 5 kΩ by rubbing the skin with EEG preparation gel prior to electrode placement. An ECG electrode was placed on the left clavicle to record heart rate.

### 2.6 Data analysis for all datasets

EEGLAB (Delorme & Makeig, 2004) and custom-made scripts were implemented in MATLAB (Mathworks) to filter, epoch, and reject EEG traces, as well as fit the n-NRF to the EEG data. The steps in this pre-processing pipeline match those used for the analyses of the Pilot Data for power calculations presented above, except where explicitly stated otherwise. The EEG processing procedures were identical for the datasets, with the exception of downsampling the data from 2048 Hz to 2000 Hz for the Exeter dataset.

The procedures and parameters in the EEG processing pipeline are as follows:

#### 2.6.1 Filtering and epoching

Raw EEG data from the Cz electrode (continuous data for the Oxford and Exeter dataset and 4-s epochs for the UCL dataset) were loaded into MATLAB using EEGLAB's loadcurry plugin or Brainstorm (Tadel, Baillet, Mosher, Pantazis, & Leahy, 2011). If not already recorded at 2000 Hz, data were downsampled to 2000 Hz using the *resample* function of EEGLAB (Delorme & Makeig, 2004) to allow compatibility with the n-NRF. Next, data were filtered. The Oxford pilot data were filtered between .50 Hz and 30 Hz (pass-band edges) using Hamming-windowed sinc FIR filters.^8^ The UCL and Exeter data were filtered between 1 Hz and 30 Hz (pass-band edges, Hamming-windowed sinc FIR filters, high-pass −6 dB cut-off = .5 Hz, transition bandwidth = 1 Hz, low-pass −6 dB cut-off = 33.75 Hz, transition bandwidth = 7.5 Hz) and a notch filter at 50 Hz.^9^ Data were then epoched with 1000 ms before and after each stimulus annotation for outcome neutral checks,^10^ and 500 ms before and 1000 ms after each stimulus annotation of interest for analysis for each hypothesis. Epochs were baseline-corrected to the pre-stimulus mean.

#### 2.6.2 Rejection procedure

EEG traces were assessed in a time-window of −500 ms to +1000 ms around the stimulus marker. Traces with signal amplitude greater than ±150 μV were rejected. Traces with a greater than 100 μV difference between maximum and minimum baseline amplitude in the pre-stimulus period were rejected. Traces with visually apparent artefact falling within these pre-defined amplitude bounds were also rejected (e.g., gross movement artefact, muscle artefact, line noise or other technical artefact).

#### 2.6.3 Projection of the n-NRF

The n-NRF was projected onto all un-rejected Cz EEG traces in a time-window 400–700 ms after the stimulus. Each individual trial was first Woody filtered to the n-NRF with a maximum jitter of ±100 ms in the n-NRF fit time-window to allow for individual differences in stimulus response latency (Woody, 1967). The result of this n-NRF projection is a scaling factor which represents the magnitude of noxious-evoked response for each trial, also referred to as the n-NRF magnitude, or n-NRF coefficient (previously referred to as the template magnitude or coefficient).

### 2.7 Blinding procedure

The same analyst pre-processed the EEG data and analysed results. The authors M. Aspbury and R. Mansfield were not involved in any of the prior published studies which are being tested for reproducibility in this study. The UCL data was not viewed or accessed by the authors and blinded data analysts, M. Aspbury and R. Mansfield, prior to in principle acceptance of the protocol, although aspects of the data were observed and previously analysed for separate research concerning premature infants’ discriminable behavioural, physiological, and brain responses to noxious and non-noxious stimuli by authors *M. van* der Vaart, C. Hartley, L. Baxter, M. M. Cobo, S. Fitzgibbon and R. Slater (van der Vaart et al., 2022). This included 1) visualisation of raw EEG for control heel lance and heel lance across a subset of the dataset (infants aged 28–40 weeks PMA) 2) extraction of the main waveforms in the EEG response data; 3) projection of the n-NRF to the EEG data for inclusion in a machine learning model. However, the metadata are available to view as supplementary material, under isa-tab metadata, with the publication (https://doi.org/10.1038/sdata.2018.248), which was accessed by the author M. Aspbury on October 12th 2021 to estimate sample sizes for the planned analysis outlined in this report.

### 2.8 Statistical analyses

**Fig. 2:**
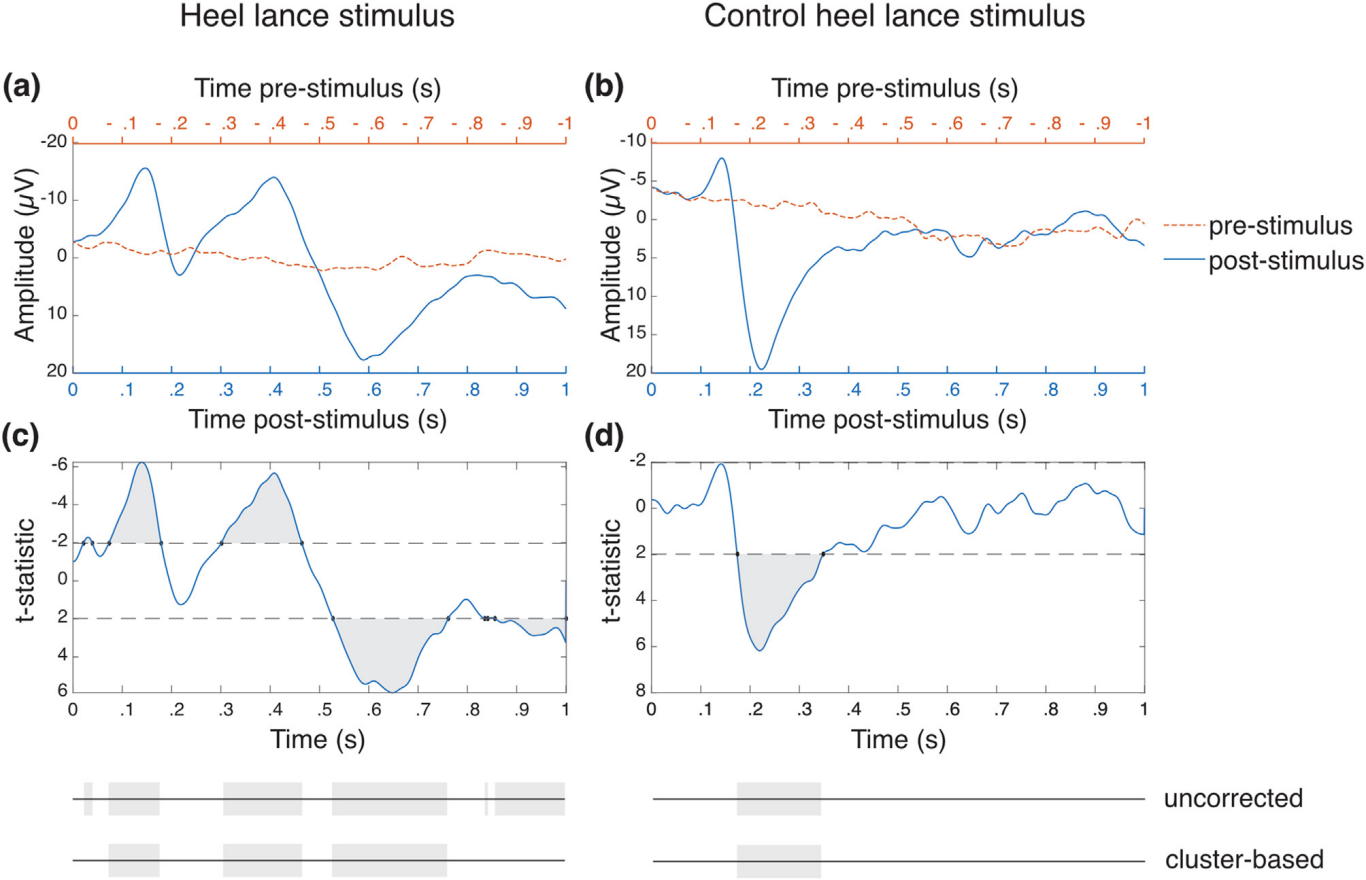
Outcome neutral criteria checks for all eligible data from the UCL and Exeter datasets. **(a)** Heel lance stimulus EEG (*n* = 135). **(b)** Control heel lance stimulus EEG (*n* = 91). **(c)** Heel lance non-parametric cluster analysis results. **(d)** Control heel lance non-parametric cluster analysis results. Fig. 2a and b shows stimulus EEG trial averages overlayed for the 1-s post-stimulus (blue) and 1-s pre-stimulus (orange), where heel lance (a) and control heel lance (b) stimuli are at 0 s. Fig. 2c and d shows non-parametric cluster analysis performed between all participant post- and pre-stimulus EEG traces, and the shaded regions are identified regions of difference before the cluster-based correction. Clusters which are significantly different after cluster-based correction are shaded at the bottom of the figure (*p* < .05). Both stimulus conditions show a significant post-stimulus response compared to the pre-stimulus activity.

**Fig. 3:**
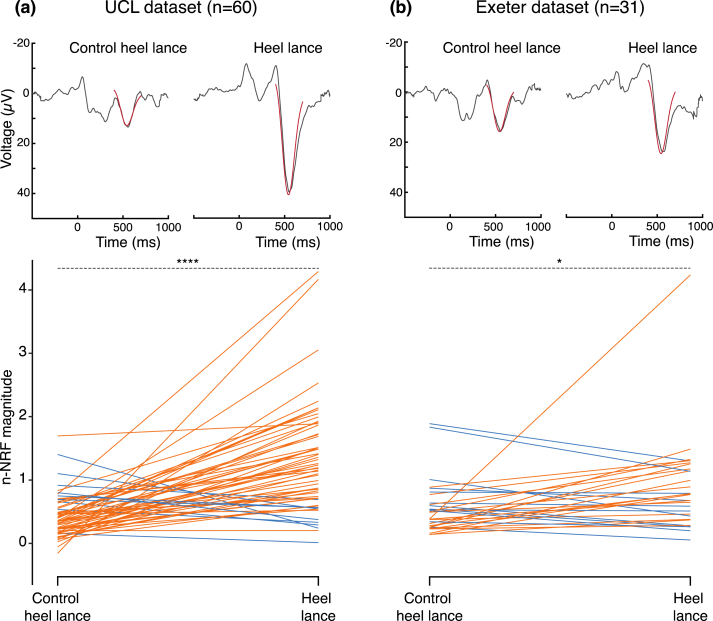
Results of Hypothesis 1 for the **(a)** UCL (*n* = 60) and **(b)** Exeter (*n* = 31) datasets. Top panel: mean Woody filtered EEG traces for control heel lance data and heel lance data (grey) with the n-NRF fitted and overlaid on the data (red). The stimulus occurred at time point 0 ms. Bottom panel: n-NRF magnitudes in response to control heel lance and heel lance at each site, paired by participant. Data is represented in orange for participants whose heel lance n-NRF magnitude was greater than their control heel lance n-NRF magnitude, and in blue for participants whose heel lance n-NRF magnitude was lower than their control heel lance n-NRF magnitude. Asterisks demonstrate significant differences between response magnitudes to the control lance compared to the heel lance in both sites (∗*p* < .05; ∗∗∗∗*p* < .0001), assessed using a one-sided paired *t*-test to evaluate whether the evoked response to the heel lance was greater than the evoked response to the control lance.

**Fig. 4:**
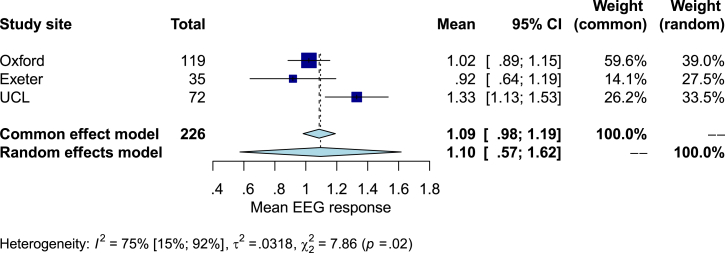
Forest plot showing the pooled mean and 95% confidence interval for the mean magnitude of the n-NRF to a heel lance at each site. The heterogeneity results are displayed below the forest plot.

**Fig. 5:**
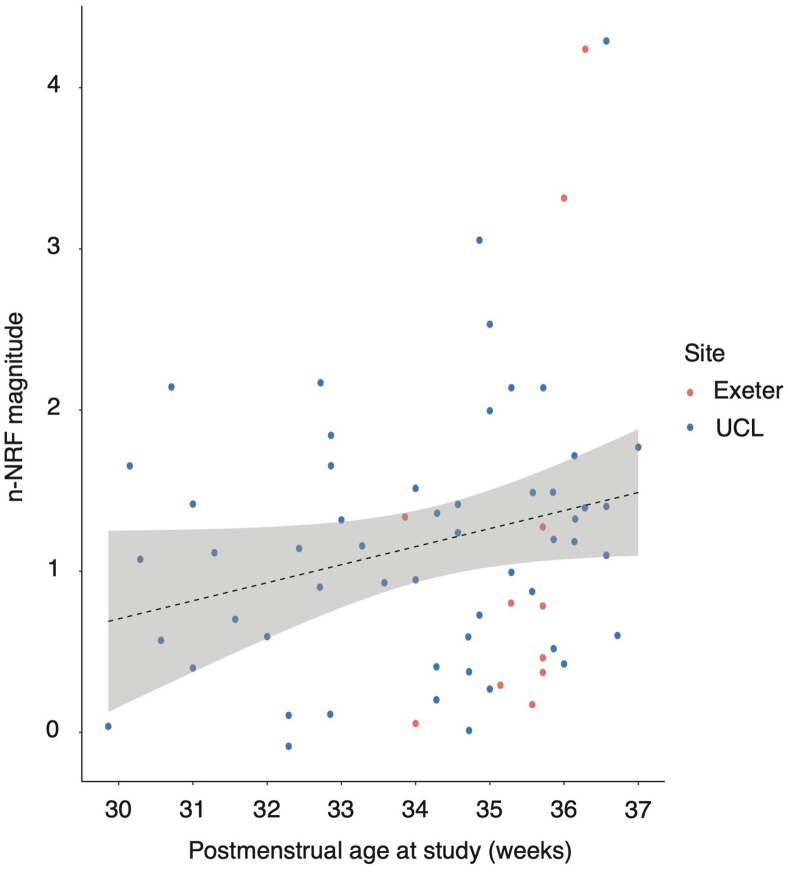
Scatter plot showing the magnitude of the n-NRF in response to a heel lance versus postmenstrual age at time of the study. UCL dataset B: *n* = 54; Exeter dataset B: *n* = 11. The linear least squares regression line fit to the data with 95% confidence interval is shown. The partial Pearson's correlation adjusted for site is *R* = .24, *p* = .03. Mean heel lance n-NRF magnitude = 1.18, standard deviation = .91. The infants were aged 23.3–35.9 weeks GA at birth (mean = 32.1 weeks), and 29.9–37.0 weeks PMA at study (mean = 34.3 weeks).

The statistical analyses for quality control outcome neutral criteria and each hypothesis we have assessed for reproducibility are presented individually below. The core scripts necessary to do the analyses and create Fig. 2, Fig. 3, Fig. 4, Fig. 5 are available (10.5281/zenodo.12682219). The code underpinning the preprocessing and response magnitude extraction are available via prior publications (Hartley et al., 2017), and are based primarily on standard EEGLAB functions (Delorme & Makeig, 2004). The code deriving results from magnitudes are available (10.5281/zenodo.12682219). Source data for Fig. 2, Fig. 3, Fig. 4, Fig. 5 arising from the Exeter dataset are available (10.5281/zenodo.12682219).

#### 2.8.1 Outcome neutral criteria

We checked Outcome Neutral Criteria in all infant EEG data, before testing our hypotheses. These conditions are independent of the hypotheses and are designed as a quality control step to ensure that our hypotheses are tested with valid data, and that data are not faulty due to technical or other error. The following checks were performed for this purpose. For Outcome Neutral Criteria involving the control heel lance stimulus data, we only performed these checks for the included control heel lance data used to test Hypothesis 1, as the other hypotheses only rely on heel lance data. All included heel lance data were combined to check for outcome neutral criteria across datasets. The outcome neutral criteria are based on the premise that if EEG data quality is sufficient, evoked activity should be present in response to both the heel lance and the control heel lance.

We performed a within-trials non-parametric cluster-based permutation analysis, in all included EEG traces recorded at the Cz electrode, to test for significant differences between the period 1-s pre-stimulus and 1-s post-stimulus (Maris & Oostenveld, 2007). We used pre- and post-stimulus EEG epochs of equal length (1 sec each) in order to use paired statistics. EEG traces were first processed up to and including the filtering stage described in Section 2.6, without Woody filtering. For each infant, the difference between the pre-stimulus and the post-stimulus data was computed, and t-statistics were calculated at each timepoint. Clusters of timepoints with t-statistics greater than the 97.5th percentile of the t-distribution were identified. Cluster mass (defined as the sum of the t-statistics in the cluster) were calculated and were assessed for significance using 10,000 random permutations of the data (significance threshold *p* < .05). We expected to see significant clusters of difference between pre- and post-stimulus epochs for both control heel lance and heel lance stimuli separately, which would demonstrate that there are stimulus-evoked signals in the EEG in each stimulus condition.

#### 2.8.2 Analysis plan to test Hypothesis 1: assessing the generalisability of the n-NRF to discriminate noxious and innocuous activity

Hypothesis 1 The magnitude of noxious-evoked brain activity measured using the n-NRF should be greater following a noxious heel lance compared with a non-noxious control heel lance procedure in infants 34.0–43.0 weeks PMA. Thus, we hypothesised a significantly larger n-NRF magnitude in response to the heel lance relative to the non-noxious control heel lance in infants 34.0–43.0 weeks PMA, assessed using a paired *t*-test.

To test Hypothesis 1, we conducted two studies:

(1) We retrospectively analysed all data available in the UCL Dataset for the inclusion criteria for this hypothesis (outlined in Section 2.2.1: UCL Dataset A) which we estimated to be *n* ≈ 88 from the public metadata.
(2) We included data for all infants recruited from The Royal Devon & Exeter Hospital (*n* = 40) since 04/06/2021, with inclusion criteria outlined in Section 2.2.1: Exeter Dataset A.

Following data inclusion (Section 2.2) and EEG processing procedures (Section 2.6), for each dataset we performed a paired one-tailed *t*-test to test that the heel lance responses were greater than control heel lance responses, as measured by the n-NRF. We prespecified that if the heel lance response was significantly greater than control heel lance in each dataset (*p* < .05) then the hypothesis would be confirmed, and the result that the n-NRF discriminates between a noxious heel lance and a non-noxious control heel lance would be reproduced across multiple centres. Otherwise, we planned to report that we were unable to reject the null hypothesis and failed to reproduce this result.

#### 2.8.3 Analysis plan to test Hypothesis 2: assessing the consistency of the n-NRF magnitude

Hypothesis 2 The group average magnitude of the noxious-evoked brain activity measured with the n-NRF should have a magnitude of 1.0 in response to a heel lance in newborn term-aged infants. Thus, we hypothesised that the average n-NRF magnitude in response to the heel lance is equivalent to 1.0, assessed using the magnitude confidence interval and pre-defined equivalence bounds.

To test Hypothesis 2, we calculated the mean and 90% confidence interval for heel lance response in the UCL dataset (Section 2.2.1: UCL dataset A). The confidence interval was calculated by bootstrap resampling using the boot library in R (Canty & Ripley, 2021; Davison & Hinkley, 1997). The Bias Corrected and accelerated (bCa) method was used (Efron, 1987), and 10,000 bootstrap samples were taken. If the mean and 90% CI are within the bounds .8–1.2, which is ±20% around 1.0, we prespecified that the mean heel lance response could be considered equivalent to 1.0 in an independent dataset according to FDA guidelines (see Section 2.4.2), and that we would then confirm this hypothesis. If the 90% confidence interval spread beyond these bounds (outside .8–1.2) then we prespecified that we would be unable to conclude whether the mean heel lance is equivalent across sites, and this hypothesis would be unconfirmed.

The AIPE sample size planning presented in Section 2.4.2 suggested this hypothesis test required us to recruit *n* = 80 infants. It was not feasible to plan to recruit this sample size at the Exeter site during the proposed study period, hence we planned for this study to be conducted in the retrospective UCL dataset (expected sample size from UCL was *n* ≈ 88; data inclusion detailed in Section 2.2.1: UCL Dataset A). However, we have also reported results of the mean and 90% CI for heel lance response in the smaller (*n* ≤ 40) Exeter Dataset A for transparency and completeness, but this may not be conclusive due to the smaller sample size.

#### 2.8.4 Analysis plan to test Hypothesis 3: assessing whether the magnitude of the n-NRF following a heel lance increases with PMA in premature infants

Hypothesis 3 The magnitude of the noxious-evoked brain activity measured with an EEG template approach in response to a heel lance has been reported to increase in premature infants with PMA up to 37.0 weeks. Thus, we hypothesised a statistically significant positive correlation between PMA and the n-NRF magnitude in response to the heel lance, assessed using a Pearson correlation test.

We prespecified that if Hypothesis 1 was unconfirmed then we would lack the basis to test Hypothesis 3, as we first need to assume that we are measuring the magnitude of noxious-evoked brain activity to a heel lance. If hypothesis 1 was confirmed, then we planned to proceed with the analysis plan to test this third hypothesis.

Hypothesis 3 was designed to assess the reproducibility of a biological finding of age-related change in noxious-evoked EEG activity following a heel lance using the n-NRF measure, as opposed to the generalisability of n-NRF characteristics across multiple sites. Therefore, to test Hypothesis 3 we planned to combine all data in the retrospective UCL Dataset (we estimated *n* ≈ 56 would be eligible in the UCL dataset) and the prospective Exeter Dataset (*n* ≤ 40), which match inclusion criteria as outlined in Section 2.2.1: UCL Dataset B and Section 2.2.1: Exeter Dataset B. The majority of the combined data was sourced from the retrospective UCL dataset, as it is a larger dataset than the Exeter sample. Unlike Hypothesis 1 and 2, we did not intend to recruit additional infants in Exeter to specifically test Hypothesis 3. We planned to include any available data from the prospective Exeter dataset that matched the criteria to test this hypothesis. Data from UCL alone was predicted to at least meet the calculated sample size requirement of *n* = 38 infants to test this hypothesis (see Section 2.4.3). We therefore expected to exceed the required sample size.

To test Hypothesis 3, we combined retrospective UCL and Exeter data and performed a one-sided Pearson correlation analysis testing for a positive correlation between PMA and heel lance n-NRF magnitude. We included a confound variable for the data site (UCL or Exeter) to account for any differences in response magnitude between different sites. The 95% confidence interval for the one-sided correlation was calculated using bootstrap resampling with 10,000 bootstrap samples. We prespecified that if the Pearson correlation is positive between PMA at study and heel lance n-NRF response with *p*-value <.05, accounting for the site confound variable, then this hypothesis would be confirmed. Otherwise, we would report that we are unable to reject the null hypothesis and had failed to reproduce this result.

Table 1 presents a summary of the data required from the UCL and Exeter sites for each of the stages of analysis planned as part of this study.

#### 2.8.5 Methods for exploratory meta-analysis (unregistered analyses)

We performed an exploratory meta-analysis of mean n-NRF heel lance response magnitudes at each site to provide context to the hypothesis 2 results. Using the mean and standard deviation of the magnitude of the EEG response to the noxious heel lance stimulus for each site (Oxford pilot dataset A, Exeter dataset A and UCL dataset A), we performed a meta-analysis of means using the meta package in R (Balduzzi, Rücker, & Schwarzer, 2019; R Core Team, 2022). The generic inverse variance method was used to pool the data. It is likely that both sampling error and between site differences may account for inter-site variation in mean magnitudes: therefore, a random effects model would be ideal. However, with only three sites, the random effects model was deemed inappropriate: it is frequently cited that a minimum of five factor levels is required for a meaningful random effects model (Jackson & Turner, 2017). Therefore, both a common effects model and the random effects model are reported for completeness. A Knapp-Hartung adjustment was applied to the random effects model (Knapp & Hartung, 2003; Sidik & Jonkman, 2002). Restricted maximum likelihood was used to estimate inter-study heterogeneity (Viechtbauer, 2005).

#### 2.8.6 Software used for generating figures

Fig. 2 and the upper panel of Fig. 3 were created in MATLAB (Mathworks). Fig. 4, Fig. 5 and the lower panel of Fig. 3 were created in R (R Core Team, 2022) using the dabestr (Ho, Tumkaya, Aryal, Choi, & Claridge-Chang, 2019), ggplot2 (Wickham, 2016) and meta (Balduzzi et al., 2019) packages.

## 3 Results

### 3.1 Participants

The full UCL dataset included 112 infants and the full Exeter dataset included 40 infants. After considering eligibility criteria for each hypothesis, the total number of infants included in each hypothesis from UCL were *n* = 60 (Hypothesis 1), *n* = 72 (Hypothesis 2), and *n* = 54 (Hypothesis 3). From the Exeter dataset, *n* = 31 (Hypothesis 1), *n* = 35 (Hypothesis 2) and *n* = 11 (Hypothesis 3) were included (Fig. 1). The demographic details of the UCL and Exeter datasets used for each hypothesis are detailed in Table 2.

**Table 2:** Demographic details for UCL and Exeter Datasets A and B. All infant data in Datasets A were used for Hypothesis 2. For Hypothesis 1, only the infants in Datasets A that had a control lance and a heel lance that met EEG quality criteria were included (UCL *n* = 60; Exeter *n* = 31). For Hypothesis 3, all infant data in Datasets B were used. Data are presented as median (interquartile range) or number (%).

	UCL Dataset A	UCL Dataset B	Exeter Dataset A	Exeter Dataset B
Number of infants	72	54	35	11
Gestational age at birth (weeks)	36.6 (35.1, 39.3)	32.7 (29.9, 34.8)	37.4 (36.1, 39.9)	34.1 (32.9, 35.3)
Postmenstrual age at time of study (weeks)	37.4 (35.8, 39.8)	34.6 (32.7, 35.7)	38.1 (36.8, 40.4)	35.7 (35.2, 35.7)
Postnatal age at time of study (days)	5 (2,6)	6 (5, 13.8)	2 (1, 5)	6 (4.5, 11)
Birthweight (g)	2625 (2185, 3453)	1780 (1290, 2232)	3200 (2417, 3710)	1937 (1676, 2220)
**Sex**				
Male	35 (49%)	26 (48%)	16 (46%)	5 (45%)
Female	37 (51%)	28 (52%)	19 (54%)	6 (55%)
**Mode of delivery**				
Normal vaginal delivery	21 (29%)	14 (26%)	13 (37%)	1 (9%)
Assisted vaginal ventouse/forceps or breech	14 (19%)	13 (24%)	4 (11%)	0
Emergency C-Section	23 (32%)	19 (35%)	11 (31%)	8 (73%)
Elective C-Section	14 (19%)	8 (15%)	7 (20%)	2 (18%)
Apgar score at 1 min	9 (7, 9)	7.5 (6, 9)^a^	8 (7, 9)^a^	7 (6, 9)
Apgar score at 5 min	9 (9, 10)	9 (8.75, 10)^a^	9 (8, 10)^a^	8 (7.5, 9)
Apgar score at 10 min	Not available	Not available	9 (9, 10)^b^	9 (9, 9.25)^a^
**Ventilation at time of study**				
Self-ventilating in air	71 (99%)	41 (76%)	34 (97%)	9 (82%)
Low flow	1 (1%)	1 (2%)	1 (3%)	0
High flow or CPAP/BiPAP	0	12 (22%)	0	2 (18%)
Estimated number of prior heel lances	7.5 (4, 13)	16 (12, 25.5)	2 (1,5)	1 (1, 4.5)

Abbreviations: C-Section – Caesarean Section; CPAP – continuous positive airway pressure; BiPAP – Bilevel Positive Airway Pressure.

a Apgar scores not recorded for up to four infants.

b Apgars not recorded for 23 infants

### 3.2 Outcome neutral criteria in UCL and exeter datasets

All eligible data obtained from UCL and Exeter (see Fig. 1) were combined to test for outcome neutral criteria. Outcome neutral criteria results for control heel lance and heel lance EEG data show that there are significant clusters of activity identified in the post-stimulus EEG when compared to the pre-stimulus EEG for both the control and noxious heel lances (Fig. 2). Therefore, the data from both UCL and Exeter satisfied the outcome neutral criteria, justifying subsequent hypothesis testing.

The outcome neutral checks for the heel lance stimulus data (*n* = 135) revealed three significant clusters: (i) a negative deflection between 74 and 179 ms (*p* = .03); (ii) a negative deflection between 302 and 465 ms (*p* = .008); (iii) and a positive deflection between 528 and 762 ms (*p* = .0006) (Fig. 2c). The outcome neutral checks for the control heel lance stimulus data (*n* = 91) showed one significant positive deflection between 175 and 349 ms (*p* = .003) (Fig. 2d). The waveforms displayed here match the noxious-evoked and non-noxious early potential waveforms published previously (Fig. 2a and b) (Cobo, Green, et al., 2022; Fabrizi et al., 2011; Hartley et al., 2017; Slater, Worley, et al., 2010; Verriotis et al., 2016).

### 3.3 Hypothesis 1 Results: noxious-evoked brain activity is greater following a noxious heel lance compared with a non-noxious control heel lance

The first hypothesis stipulated that the magnitude of the stimulus-evoked brain activity (the n-NRF) would be greater following a noxious event (heel lance) compared to a non-noxious event (control heel lance) in infants aged 34.0–43.0 weeks PMA. This hypothesis was tested in the UCL Dataset A and Exeter Dataset A separately.

From UCL Dataset A, we included heel lance and control lance EEG data for 60 infants (Fig. 1a). The mean magnitude of the n-NRF to control heel lance was .42 (95% CI .33–.50). The mean magnitude of the n-NRF to heel lance was 1.30 (95% CI 1.09–1.52). The difference in mean response between the heel lance and control heel lance was .88 (95% CI: .64–1.13; t-statistic = 7.32; *n* = 60, *p* < .0001; Fig. 3a).

From Exeter Dataset A we included heel lance and control heel lance EEG data for 31 infants (Fig. 1b), which was lower than the estimated required sample size of *n* = 35. The mean magnitude of the n-NRF to control heel lance was .55 (95% CI .40–.70). The mean magnitude of the n-NRF to heel lance was .86 (95% CI .59–1.14). The difference in mean response between the heel lance and control heel lance was .31 (95% CI: .02–.61; t-statistic = 2.15, *n* = 31, *p* = .02; Fig. 3b).

Therefore, Hypothesis 1 is confirmed in both datasets.

### 3.4 Hypothesis 2 Results: average noxious-evoked EEG activity in response to the heel lance was not demonstrated to be equivalent to a value of 1.0

Hypothesis 2 posited that the mean magnitude of the noxious-evoked brain activity to a heel lance is equivalent to 1.0 for infants at 34.0–43.0 weeks PMA. To confirm Hypothesis 2 the confidence interval for each dataset had to lie between .8 and 1.2 (width: .4). We pragmatically used an AIPE analysis to estimate that a sample size of *n* = 73 would be required to obtain a confidence interval width of .4 or narrower around the mean magnitude (see Section 2.4.2).

From UCL, 72 infants were eligible for inclusion giving adequate power to test Hypothesis 2 (Dataset A) (Fig. 1a). The mean heel lance n-NRF magnitude was 1.33 (90% bootstrap CI 1.18–1.52; interval width = .34). From Exeter, 35 infants were eligible for inclusion (Dataset A, Fig. 1b), which did not provide adequate power. The mean heel lance magnitude was .92 (90% bootstrap CI .74–1.22; interval width = .48).

As the confidence intervals for both datasets contain values that lie outside the range from .8 to 1.2, we cannot confirm that the group average heel lance-evoked magnitude is equivalent to 1.0 in either dataset.

### 3.5 Exploratory meta-analysis (unregistered analyses): average noxious-evoked EEG activity in response to the heel lance shows inter-site variability

To provide context to the findings from Hypothesis 2 and to better understand the influence of site on the result, we explored the effect of pooling the Oxford, UCL and Exeter datasets and performed a meta-analysis of the mean magnitude of the n-NRF to the noxious heel lance at each site (Section 2.8.5). In the Oxford dataset (*n* = 119) the mean magnitude was 1.02, and standard deviation (sd) was .75 (Supplementary Appendix A, Section A1.3.4). In Exeter, the mean magnitude was .92 (sd .84), and in UCL the mean magnitude was 1.33 (sd .88). The common effects model pooled mean was 1.09 (95% CI .98–1.19), but the chi-squared test for heterogeneity was statistically significant (*X*^2^ = 7.86, *p*-value = .02) indicating that not all sites share a common effect size (Fig. 4). Additionally, the proportion of total variance that reflects true between-site heterogeneity in effect size (I^2^) was 75%, which is typically interpreted as high. This requires the use of a random effects model to estimate the overall mean effect size across sites, which was 1.10 (95% CI .57–1.62), but, with such few sites and high heterogeneity, the meta-analysis lacks power (Jackson & Turner, 2017). These exploratory meta-analysis results complement our equivalence test results: the mean heel lance-evoked magnitude has significant heterogeneity among sites, and the mean heel lance-evoked magnitude in the Exeter and UCL sites were not demonstrated to be equivalent to 1.0.

### 3.6 Hypothesis 3 results: the mean magnitude of the noxious-evoked EEG response is positively correlated with PMA at time of study

We aimed to reproduce the finding that the magnitude of noxious-evoked brain activity in response to a heel lance increases with PMA in independent datasets (Section 2.8.4). As planned, data from both UCL Dataset B (*n* = 54) and Exeter Dataset B (*n* = 11) were combined for this hypothesis, which exceeded the pre-specified sample size (*n* = 38) that was required to test this hypothesis (Section 2.4.3).

The one-sided partial Pearson correlation between the heel lance response magnitude and PMA, adjusted for site was *R* = .24 (95% CI .06–1.00, *p* = .03), confirming Hypothesis 3 (Fig. 5).

## 4 Discussion

This pre-registered reproducibility study set out to test three hypotheses related to the use of the n-NRF, which is an EEG-based measurement designed to quantify neonatal brain responses to noxious stimulation. Using data that was collected at two different sites (UCL and Exeter), we confirmed that the magnitude of the n-NRF is significantly greater to a noxious heel lance stimulus than to a non-noxious control stimulus (hypothesis 1). Using data from UCL and Exeter combined, we reproduced the finding that the magnitude of the n-NRF to a heel lance increases with PMA from 30 to 37 weeks (Hypothesis 3). Hypothesis 2, which stipulated that the magnitude of the n-NRF would be equivalent to 1.0 across sites, was not confirmed. Exploring this further, we observed that there was significant heterogeneity between sites.

### 4.1 Hypothesis 1

We have shown that the n-NRF magnitude distinguished noxious-evoked brain activity from non-noxious evoked brain activity in two external datasets, since the mean magnitude obtained from the noxious stimulus was significantly greater than that obtained from the non-noxious stimulus (Hypothesis 1). The reproducibility of this finding helps further establish the construct validity of the analysis method, by supporting a variation on “known groups validity” (Baxter et al., 2023). Here, the known groups were instead known stimuli, with one skin-breaking noxious stimulus and one non-skin-breaking non-noxious stimulus.

Previous data published by our group has consistently demonstrated the construct validity pertaining to the use of the n-NRF. For example, when the n-NRF is applied to EEG activity evoked by a variety of non-noxious stimuli (tactile, auditory and visual) its magnitude is not significantly different compared to when it is applied to background EEG activity (Hartley et al., 2017). In contrast, when the n-NRF was used to quantify brain activity evoked by a noxious event, its magnitude was significantly greater than the n-NRF magnitude observed in background activity and non-noxious stimulus data (Hartley et al., 2017). We have also previously demonstrated that the magnitude of the response decreases following the administration of topical local anaesthetic and paracetamol (Cobo et al., 2021; Hartley et al., 2017). In addition, we have previously demonstrated convergent validity, as the n-NRF magnitude has a significant, albeit moderate, correlation with the commonly-used Premature Infant Pain Profile – Revised (PIPP-R) score (Stevens et al., 2014) that has been developed to quantity pain experience in infants (Hartley et al., 2017).

These findings, alongside the reproducibility of Hypothesis 1 at the independent Exeter and UCL sites, support the use of the n-NRF as an outcome measure in clinical trials that aim to quantify the analgesic efficacy of pharmacological or non-pharmacological interventions for acute painful clinical procedures.

### 4.2 Hypothesis 2

The n-NRF is scaled to give an average magnitude of 1.0 in response to a heel lance in term infants (Hartley et al., 2017), and our pilot analysis of infants from 34.0 to 43.0 weeks PMA showed the average n-NRF magnitude was equivalent to 1.0 (Supplementary Appendix A). Therefore, for Hypothesis 2, we tested the equivalence of the scaling of the n-NRF across sites by testing whether the mean heel lance-evoked n-NRF magnitudes in the Exeter and UCL sites (including infants aged from 34.0 to 43.0 weeks PMA) are equivalent to a value of 1.0, where equivalence to 1.0 includes values in the range .8–1.2.

In both instances, the 90% CIs included null hypothesis values of less than .8 (Exeter) or greater than 1.2 (Exeter and UCL), thus failing to demonstrate equivalence to 1.0. It is unclear whether this result is a true negative due to non-equivalence of the n-NRF to 1.0 at these sites, or a false negative due to underpowering.

Here, it is important to consider the pragmatic approach used in the sample size calculations for this hypothesis. Since a 90% CI lying within 1.0 ± .2 would be in line with the FDA guidelines for bioequivalence studies (Section 2.4.2), we used an AIPE analysis to calculate the estimate sample size needed to achieve a CI width of .4. As equivalence testing requires the full 90% CI of the mean estimate to lie within the range of interest (in this case .8–1.2; width = .4), a CI width of .4 represents the minimum precision of the mean estimate that would be needed to reject the null hypothesis. This gave a required sample size of *n* = 73. In contrast, the estimated sample size required for achieving 90% power in testing the equivalence of the mean to 1.0 (Lakens, 2017), as calculated using the TOSTER package in R (Caldwell, 2022), would have been 154, which far exceeds the size of the UCL dataset. Therefore, substantially larger datasets would be required to help to disambiguate the inconclusive result for Hypothesis 2. Our exploratory meta-analysis of the raw mean n-NRF magnitudes obtained at three sites (Oxford, Exeter and UCL) indicated that there was statistically significant heterogeneity between sites. While there is an important difference between statistical significance (assessed in our meta-analysis) and substantive significance (assessed in our equivalence test), the statistically significant heterogeneity in mean n-NRF magnitude between sites is a novel and important observation, which raises two important considerations for future studies.

The first consideration surrounds the source of inter-site heterogeneity. This is likely a combination of non-biological effects, such as differences in EEG recording and heel-lance equipment and researcher effects (e.g. variability in electrode placement and procedure technique) and biological effects, driven by issues such as a lack of matching between study samples and differences in demographics. Unfortunately, given the small number of sites and lack of recorded variables potentially relevant to explaining inter-site variability, sub-group or meta-regression analyses were not possible. Future areas of research include to understand biologically-driven reasons for the inter-infant variability in the magnitude of the response to the heel lance stimulus, which may be related, for example, to infection (Cobo, Green, et al., 2022), mode of delivery (Kasser et al., 2019), stress (Jones et al., 2017), age (Green et al., 2019; Hartley et al., 2016; Schmidt Mellado et al., 2022) or sex (Verriotis et al., 2018), since a deeper understanding of the importance of these factors may aid in adequate stratification during randomisation for clinical trials of analgesics in the future.

The second consideration raised by our exploratory meta-analysis results is how to appropriately address the observed inter-site heterogeneity in the design of future multi-site studies using the n-NRF as an outcome measure. Not controlling for site effects could have a detrimental impact on trial sensitivity, as subtle analgesic efficacy (or harm) effects could be masked by substantial site effects. Therefore, this finding should inform the use of a site-based confound variable when the n-NRF is used in multi-centre trials. In addition, it should be highlighted that for future single-site studies, expected normative ranges at other sites may differ from the values obtained in the original Oxford dataset (Hartley et al., 2017). Site-specific normative values should be generated if researchers are interested in studying absolute n-NRF values at their site.

Findings in the field of neuroimaging are often influenced by differences in equipment and set up (Melnik et al., 2017). As methods for harmonising and standardising neuroimaging data acquired in different sites improve, exploring equivalence of the n-NRF magnitude at different sites will likely become more achievable as has been shown across other disciplines (Dinsdale, Jenkinson, & Namburete, 2021; Farzan et al., 2017; Prado et al., 2022). The n-NRF was designed for use at the group level. The site heterogeneity in our analysis underlines that standardisation and harmonisation methods would need to be developed before the n-NRF analysis methodology can be considered for single-patient diagnostic purposes, where interpretation of the magnitude at the patient-level is required.

### 4.3 Hypothesis 3

The results of Hypothesis 3 confirmed that the n-NRF magnitude increased with PMA in premature infants up to 37 weeks PMA at the time of study. There is value in reproducing biological correlates with the n-NRF magnitude to confirm that the behaviour of the n-NRF is consistent across populations. Confirming this hypothesis reinforces the consistency and generalisability of this analytical approach.

The current study was not designed to investigate the underlying neurological mechanism of this correlation. As such we cannot draw conclusions on whether the change of n-NRF magnitude across age reflects changes in, for example, brain structure, nociception, experience, or a combination of any of these factors. Our results do indicate the importance of taking PMA into account in the design of neonatal analgesic studies. It is well-known that noxious-evoked brain responses change throughout the preterm period (Fabrizi et al., 2011; Hartley et al., 2016), in line with ongoing anatomical and functional changes (Doria et al., 2010; Dubois et al., 2014; Hatfield, 2014). Hence, it is reasonable to think that there may be a developmental change in n-NRF magnitude in response to a heel lance. However, recent work performed within our group has shown how the morphology (rather than simply the magnitude) of noxious-evoked brain activity changes during the preterm period (van der Vaart et al., 2022), which implies a need for an age-appropriate n-NRF template for use in preterm infants below approximately 34 weeks of age.

Indeed, using an age-appropriate n-NRF that can discriminate noxious from non-noxious stimulus responses could transform the diagnosis and treatment of pain in preterm infants, in whom facial grimacing, limb withdrawal and heart rate responses have been found not to be reliably distinct between noxious and non-noxious stimulus responses (Craig et al., 1993; Cornelissen et al., 2013; Green et al., 2019).

### 4.4 Limitations

Due to time and resource constraints, we were not able to collect the Exeter data prospectively from the date of pre-acceptance of this study's protocol (protocol submitted on 25/01/2022; protocol accepted on 11/09/2022). Therefore, the Exeter dataset is formed from data collected from 04/06/2021 onwards and included data from infants who received a parental touch intervention that did not influence the magnitude of the evoked response (Cobo, Moultrie, et al., 2022; Hauck et al., 2024). However, the method of data collection was identical to that laid out in the pre-registered protocol.

While we have shown the generalisability of our findings at multiple research sites in the United Kingdom (UCL and Exeter), we cannot comment on the generalisability of these findings outside the centres included here. In order to investigate the broader generalisability of our findings, our group is undertaking an international individual participant data (IPD) meta-analysis of noxious-evoked neonatal EEG data acquired globally (Baxter et al., 2023). The current reproducibility study and the ongoing IPD meta-analysis have the potential to transform the way analgesic efficacy studies are performed, by providing evidence for a much needed and objective outcome for measuring the acute pain experience of neonates.

Finally, we set out to explore whether the mean n-NRF magnitude in response to a heel lance was equivalent to 1.0 at other sites in hypothesis 2, and we acknowledge that this requires a clear and justified definition of equivalence. However, given the challenges in pain assessment and research in neonates and the novelty of applying brain imaging methods for neonatal pain assessment, there is no precedent, let alone established consensus, known to the authors on how to define equivalence in this context. Due to the lack of any existing foundations, we based our definition of equivalence on the bioequivalence norms used by regulatory bodies for pharmacokinetic studies. We do not yet know if the equivalence range used in our analysis will map onto clinically meaningful ranges of effects. The range of values that are not substantively or clinically meaningfully different from a value of 1.0 may be wider or narrower than our chosen definition of .8–1.2. While it is easy to critique our choice of equivalence range, we believe it was a reasonable first attempt, and look forward to improving our understanding of how to define what are and are not clinically meaningful effect sizes for neuroimaging outcomes for infant pain assessment. We believe this is simultaneously one of the most challenging and important topics for infant pain research to address.

### 4.5 Conclusions

By reproducing key findings relating to the n-NRF, this study has provided evidence for the construct validity and generalisability of this approach for quantifying noxious-evoked brain activity. Nevertheless, reliability could be improved by standardising techniques and calibration of equipment across sites. This study therefore supports use of the n-NRF as a pre-defined outcome for use in clinical trials of analgesic efficacy in neonates, and our findings emphasise the importance of including a site-specific confound variable for future multi-centre trials.

There is mounting evidence supporting the construct and convergent validity of the n-NRF approach shown in our group's previous research (Cobo et al., 2021; Gursul et al., 2018; Hartley et al., 2017) and reproduced here, and we have now shown evidence of generalisability of our findings to external sites. In addition, the n-NRF approach has a key advantage over other existing tools: the n-NRF provides an objective measure for assessing acute nociception in neonates. Therefore, we strongly encourage researchers collecting acute neonatal pain-related EEG data to adopt this methodology. We believe there is now a robust case emerging to establish our n-NRF analysis methodology as a standardised approach for studying acute nociception in neonates and we would welcome collaborators to use our analysis pipeline in future observational studies or clinical trials.

## Open practices

The study in this article has earned Preregistered badges for transparent practices. The preregistered studies are available at: https://doi.org/10.17605/OSF.IO/ZY9MS

## Funding sources

This research is funded by the Wellcome Trust via a senior fellowship awarded to R.S. (Grant number 207457/Z/17/Z). The Petal trial was funded by the Wellcome Trust and by Bliss (a UK charity). The Engineering and Physical Sciences Research Council (EPSRC, Grant number EP/L016044/1) funded MA; the Wellcome Trust and Royal Society funded CH through a Henry Dale Fellowship (Grant number 213486/Z/18/Z); Bliss (a UK charity) funded LB; a Wellcome Trust collaborative award (215573/Z/19/Z) and senior fellowship (221933/Z/20/Z) funded SF. RP's Clinical Lectureship is recognised by NIHR and funded by the University of Exeter. Dr. Roshni C Mansfield, Academic Clinical Fellow, ACF-2021-13-004, is funded by Health Education England (HEE)/NIHR for this research project. The views expressed in this publication are those of the author(s) and not necessarily those of the NIHR, the University of Oxford, NHS or the UK Department of Health and Social Care. None of the funding sources had any role in study design, data collection, data analysis, data interpretation, writing the manuscript or in the decision to submit the article for publication.

## CRediT authorship contribution statement

**Marianne Aspbury:** Writing – review & editing, Writing – original draft, Visualization, Validation, Software, Methodology, Funding acquisition, Formal analysis, Data curation, Conceptualization. **Roshni C. Mansfield:** Writing – review & editing, Writing – original draft, Visualization, Validation, Methodology, Funding acquisition, Formal analysis, Data curation. **Luke Baxter:** Writing – review & editing, Visualization, Validation, Supervision, Software, Methodology, Formal analysis, Data curation, Conceptualization. **Aomesh Bhatt:** Writing – review & editing, Conceptualization. **Maria M. Cobo:** Writing – review & editing, Validation, Investigation, Data curation. **Sean P. Fitzgibbon:** Writing – review & editing, Methodology, Conceptualization. **Caroline Hartley:** Writing – review & editing, Validation, Supervision, Software, Resources, Methodology, Investigation, Funding acquisition, Data curation. **Annalisa Hauck:** Writing – review & editing, Visualization, Investigation, Data curation. **Simon Marchant:** Writing – review & editing, Software, Methodology, Data curation. **Vaneesha Monk:** Writing – review & editing, Project administration. **Kirubin Pillay:** Writing – review & editing, Data curation. **Ravi Poorun:** Writing – review & editing, Resources, Project administration, Investigation, Data curation. **Marianne van der Vaart:** Writing – review & editing, Visualization, Validation, Supervision, Software, Methodology, Investigation, Formal analysis, Data curation, Conceptualization. **Rebeccah Slater:** Writing – review & editing, Validation, Supervision, Resources, Project administration, Methodology, Funding acquisition, Formal analysis, Conceptualization.

## Declaration of competing interest

None.

## Footnotes

3 Since writing the pre-registered protocol, we have changed the terminology used for the characteristic pattern of noxious-evoked EEG activity from referring to it as the “template” to calling it the “noxious neurodynamic response function (n-NRF)”. Therefore, in this Stage 2 manuscript, we have changed the word “template” to “n-NRF” throughout, so that this manuscript is consistent with other contemporaneous work using this measure of noxious-evoked brain activity in neonates.

4 Pilot analysis results are now displayed in Supplementary Appendix A instead of in Section 3 of this paper to improve the readability of this manuscript.

5 Deviation from protocol: as stated in the main text, we were not able to recruit a fully prospective dataset from Exeter as we had initially planned. Therefore, all babies recruited and studied in Exeter are included in the results for the Exeter dataset, and the justification for this is stated explicitly in the main text of Section 2.1 above.

6 Deviation from protocol: the pre-registered protocol stated in error that for inclusion in Hypothesis 2, all babies required a heel lance and control lance procedure. Hypothesis 2 only requires babies to have a heel lance procedure, so we have included all babies that meet this criterion in the results of this manuscript to avoid unnecessary and unjustified data loss.

7 Deviation from protocol: as stated in the text, if there was insufficient information held within the UCL dataset to apply exclusion criteria, infant data was preferentially retained for analysis to preserve sample size. Infants with a diagnosis of IVH of unspecified grade and periventricular leukomalacia without further information were retained in the dataset on these grounds.

8 Edits for clarity (not a deviation): additional details are provided for reproducibility.

9 Protocol deviation: Following the processing of the Oxford pilot data, we made improvements to our research group's EEG preprocessing pipeline, which will be publicly released (in preparation). This new pipeline includes a 1 Hz low-pass filter pass-band edge as a recommended setting. We applied a 50 Hz notch filter to all data as we observed break-through 50 Hz interference.

10 Edits for clarity (not a deviation). The original protocol did not make it clear until section 2.8.1 that the outcome neutral criteria would be assessed based on 1000 ms before and after the stimulus: we have added this detail to section 2.6 for clarity of methods.
