# Supplementary material for: Establishing a standardised approach for the measurement of neonatal noxious-evoked brain activity in response to an acute somatic nociceptive heel lance stimulus
Source: Cortex. 2024 Oct;179:215–34. doi: 10.1016/j.cortex.2024.05.023 (PMC11913738; doi:10.1016/j.cortex.2024.05.023)
Supplement: Multimedia component 1 [file mmc1.docx]

**Supplementary Appendix A**

**A1. Pilot Data**

**A1.1. Participants**

**The Oxford Dataset (Pilot Data)**

Ethical approval for these data has been obtained from the NHS Research Ethics Committee as part of ongoing studies (references ﻿11/LO/0350, 12/SC/0447 and 19/LO/1085). Study metadata are stored on a secure database developed by the European company MedSciNet AB, with strict requirements for system integrity and security necessary for medical research and clinical trials. The database management conforms to GDPR standards, relevant FDA, NIH, and HL7 standards, guidelines, and recommendations (<https://medscinet.com/about.aspx>, accessed November 2021).

*Oxford Dataset A. Data inclusion for sample size planning for hypotheses 1 and 2:*

We extracted all data from our secure database of infants recruited at the John Radcliffe Hospital, Oxford, which met the following criteria: infants had EEG recorded during a test occasion and were between (inclusive) 31 and 43 weeks GA at birth and 34 and 43 weeks PMA at the time of the study. These encompass the age ranges validated in Hartley et al for both Hartley’s Study 1 of the 18 infants used in the derivation of the n-NRF and Hartley’s Study 3 of the proof of construct validity of the n-NRF in preterm neonates and infants (Hartley et al., 2017). This database extraction also excluded infants who were recruited as part of a blinded clinical trial. We exported this data from the database on 30^th^ October 2021. Following this export totalling 391 EEG recording test occasions of 371 unique infants, we then subset the data to suit our experimental paradigm. We included test occasions where both a heel lance and control heel lance procedure were recorded using EEG (n=211 test occasions).

We excluded infants and test occasions based on the following criteria: infants whose data were used in the original creation of the n-NRF (n=8), infants who were studied during a brushing or stroking intervention (n=48) (Gursul et al., 2018), infants who were included in a study investigating a kangaroo care intervention (n=3), infants with neurological conditions such as (but not limited to) HIE and IVH of any grade (n=6), test occasions where either the heel lance or control heel lance stimulus annotation was missing from the EEG record (n=5), where the EEG eventlist was incorrect or missing in storage (n=4), where the Cz electrode was not recorded (n=4), repeated samples on the database (n=2), and one of the two test occasions where an infant is studied twice (n=4). For the latter exclusion, we always included the first test occasion and excluded the second, except one infant which had multiple control heel lances on the first test occasion, so we used the second test occasion which had exactly one control heel lance and one heel lance measured. This left n=127 infants whose EEG data we pre-processed.

Following EEG pre-processing rejection steps to remove artefactual epochs (see Section 2.6), we excluded n=8 infants which didn’t have both one heel lance and one control heel lance passing EEG rejections to enable paired statistics. In another n=8 infants there were multiple heel lance and/or control heel lance measurements after EEG rejection and, in these cases, we kept the first artefact-free recording for each of the heel lance and control heel lance measurements for each infant.

This gave a final sample size of n=119 infants with both one control heel lance epoch and one heel lance EEG epoch each (see S1. Supplementary data inclusion flow diagram). This dataset comprised infants born at 31.0 – 42.4 weeks GA (mean = 38.4 weeks GA), studied at 34.1 – 43.0 weeks PMA (mean = 39.1 weeks PMA) and at 0 – 44 days PNA (mean = 4.5 days PNA).

*Oxford Dataset B. Data inclusion for sample size planning for hypothesis 3:*

For this analysis, a separate sample of neonates and infants were selected from the Oxford Database due to the different age range considered compared to the analyses for hypotheses 1 and 2. We extracted all infants from the database with EEG recordings for a clinical heel lance procedure (n=305 test occasions), on the date of export of 1^st^ November 2021. We included infants who were born at less than 36.0 weeks GA and studied at an age up to and including 37.0 weeks PMA to match the upper age limit in Schmidt Mellado et al., 2022 (n=125 test occasions). Following this, we excluded infants included in the original Schmidt Mellado et al. study (n=52 exclusions), infants with neurological conditions including HIE and IVH of grade 2 or higher (n=10), without EEG data (n=6), with a missing eventlist (n=2) or with a problem in the eventlist annotation for the heel lance such as the automatic trigger missing (n=3). We also excluded duplicate data (n=2), infants with an intervention aside from standard-care to reduce noxious response to the heel lance (n=2), and test occasions beyond the first study for infants studied more than once (n=7). This resulted in a sample of n=41 infants with one test occasion, with EEG recordings for a clinical heel lance procedure. After EEG pre-processing and rejection (Section 2.6), n=6 infant heel lance traces were rejected. In n=3 infants there were multiple heel lance measurements after EEG rejection and, in these cases, we kept the first artefact-free recording for each of the heel lance measurements for each infant.

This left n=35 infants each with one heel lance recording which were included for sample size planning for hypothesis 3 (see S2. supplementary data inclusion flow diagram). They had a GA at birth of 25.0-35.9 weeks (mean=31.1 weeks) and PMA at study of 30.1-37.0 weeks (mean=34.1 weeks), and PNA at study of 1-59 days (mean=21.2 days).

|  | **Oxford Dataset A: sample size planning hypotheses 1 and 2** | **Oxford Dataset B: sample size planning hypothesis 3** |
| --- | --- | --- |
| Number of infants | 119 | 35 |
| Gestational age at birth (weeks) | 39.4 (36.1, 40.7) | 31.3 (29.1, 33.1) |
| Postmenstrual age at time of study (weeks) | 39.7 (36.8, 41.0) | 34.1 (33.4, 35.0) |
| Postnatal age at time of study (days) | 3 (1, 5) | 24.0 (6.5, 29.5) |
| Birthweight (g) | 3510 (2770, 3923) | 1626 (1045, 2060) |
| Sex | | |
| Male | 71 (60 %) | 20 (57 %) |
| Female | 48 (40 %) | 15 (43 %) |
| Mode of delivery | | |
| Normal vaginal delivery | 42 (35.3 %) | 15 (42.9 %) |
| Assisted vaginal ventouse/forceps or breech | 34 (28.6 %) | 3 (8.6 %) |
| Emergency C-Section | 33 (27.7 %) | 14 (40.0 %) |
| Elective C-Section | 10 (8.4 %) | 3 (8.6 %) |
| Apgar score at 1 min | 9 (7, 9.8) * | 8 (4.5, 9) |
| Apgar score at 5 min | 10 (10, 10) | 9 (8, 10) |
| Apgar score at 10 min | 10 (10, 10) | 10 (9, 10) * |
| Ventilation at time of study | | |
| Self-ventilating in air | 109 (91.6 %) * | 22 (62.9 %) |
| Low flow | 5 (4.2 %) | 2 (5.7 %) |
| High flow | 4 (3.4 %) | 10 (28.6 %) |
| Estimated number of prior painful procedures | 4 (2, 8) *** | 20 (8, 42) ** |

**Table A1:** Oxford dataset demographics. Values given are median (lower quartile, upper quartile) or number (percentage). Data not documented in database for * 1 infant ** 3 infants *** 8 infants.

**A1.2. Methods**

The study protocol for the pilot data matched that described for prospective data collection in Section 2.5. Minor differences to the EEG set-up and specifics of the data analysis pipeline are described in this section.

**A1.2.1. EEG Set-up**

Pilot EEG data was acquired by the same method as described in Section 2.5.3, EEG Set-up, except that we used a SynAmps RT 64-channel headbox and amplifiers (Compumedics Neuroscan), with a bandwidth from DC - 400 Hz and a sampling rate of 2000 Hz and recorded with CURRYscan7 suite (Compumedics Neuroscan). Infants on the Oxford database either had EEG recorded with Fz as the reference electrode, or where FPz was used as the reference electrode these data were re-referenced to Fz during pre-processing.

**A1.2.2. Data Analysis**

The data analysis pipeline used was exactly as described in Section 2.6. Data Analysis for All Datasets. As planned for prospective Exeter data, continuous raw EEG data from the Cz electrode was loaded into MATLAB for processing.

**A1.3. Results from Oxford Pilot Data**

**A1.3.1. Pilot Data Outcome Neutral Criteria:**

All pilot data were combined to test for outcome neutral criteria. There are n=141 unique heel lance traces from both Oxford datasets A and B, since some participants are present in both datasets. There are n=119 control heel lance traces, which are all from Oxford dataset A, since control heel lances are not required for the third hypothesis which uses Oxford dataset B. Results for the outcome neutral criteria for each stimulus are shown in Figure A1.

There are significant clusters in the post-stimulus EEG compared to the pre-stimulus EEG for each stimulus separately, using non-parametric cluster-based analysis (Figure A1, bottom). For the heel lance stimuli (n=141), there is one significant cluster identified after correction, representing a noxious-evoked potential, from 435 – 1000 ms (p<0.0001). For the control heel lance stimuli (n=119), one significant cluster, representing a non-noxious early potential, is identified at 159 – 375 ms (p=0.005). These cluster timepoints match those reported in the literature for both the noxious potential and non-noxious early potentials (Fabrizi et al., 2011; Hartley et al., 2017; Slater et al., 2010). These data pass the outcome neutral criteria tests since there is significant post-stimulus evoked activity, not present in the pre-stimulus baseline activity, for both control heel lance and heel lance EEG traces.

**
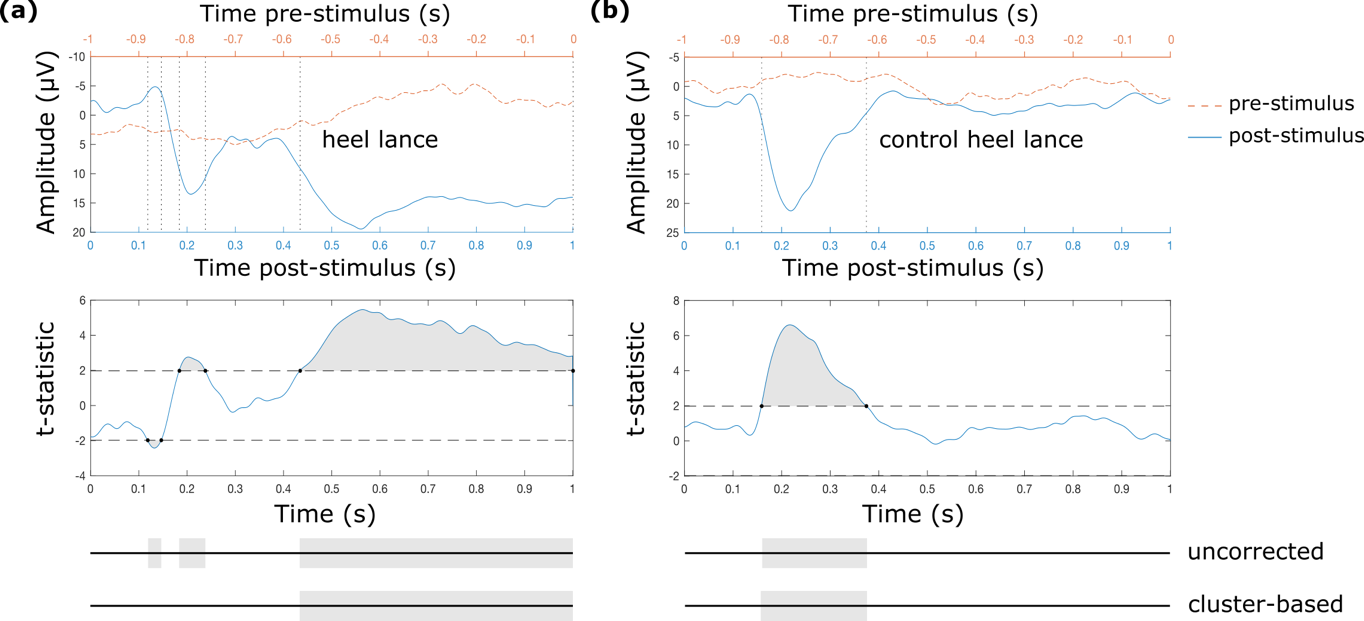
**

**Figure A1:** Neutral outcome criteria for all unique Oxford Pilot Data **(a)** heel lance stimulus EEG (n=141) and non-parametric cluster analysis results **(b)** control heel lance stimulus EEG (n=119) and non-parametric cluster analysis results. Top: stimulus EEG trial averages overlayed for the 1-second post-stimulus (blue) and 1-second pre-stimulus (orange), where stimuli are at 0 seconds. Bottom: non-parametric cluster analysis was performed between all participant post- and pre-stimulus EEG traces, and the shaded regions are identified regions of difference before the cluster-based correction. Clusters which are significantly different after cluster-based correction are shaded at the bottom of the figure (p < 0.05).

**A1.3.2. Reproducing the derivation of the n-NRF using an updated pre-processing pipeline:**

Hartley and colleagues created a brain activity “template” (the noxious neurodynamic response function, or n-NRF) that can be projected onto EEG data, which has been shown to have significantly greater magnitude in response to a noxious stimulus compared with a non-noxious control stimulus (Hartley et al., 2017). The n-NRF was scaled so that a value of 1 represents the average response evoked by a clinically-required heel lance in a term-aged neonate.

Since the original derivation and validation of the n-NRF, the pre-processing pipeline that prepares the EEG data for analysis has been updated to include refined filtering parameters (see Section 2.6). As the revised data analysis pipeline has the potential to alter the morphology of the EEG data, we reproduced the study conducted by Hartley and colleagues in the same data (n=18) using the updated analysis pipeline. The results confirm that the updated EEG data analysis pipeline did not change the original conclusions reported (Figure S2a). Using the EEG n-NRF and updated pipeline, the noxious response was still significantly greater than non-noxious response and background activity, whilst non-noxious response was not significantly different from background.

**A1.3.3. Pilot Results for Hypothesis 1:**

In the original study, the n-NRF was shown to discriminate between noxious and non-noxious stimuli in term and premature infants across multiple experiments (Hartley et al., 2017). This included a significantly larger magnitude of evoked response to a noxious heel lance compared with a non-noxious control heel lance procedure. Using a locally-acquired dataset (n=119, described in Section A1.1: Oxford Dataset A, and in Table A1) which is independent to the data used in the original n-NRF derivation (but not validation), we demonstrate that the ability of the n-NRF to discriminate between noxious heel lance and innocuous control heel lance stimuli can be observed in a larger independent dataset (Figure A2b, Table A2). This enables us to use this larger dataset for more reliable effect size estimation and sample size planning to test hypothesis 1 (Section 2.4.1). The effect size in this dataset is 0.589 (95% CI: 0.39-0.77), which is calculated by the standardised mean difference effect size for this within-participant design, i.e., the mean of differences of the heel lance and control heel lance responses divided by the standard deviation of the differences in these responses, also known as Cohen’s d_z_ (Lakens, 2013).

**A1.3.4. Pilot Results for Hypothesis 2:**

The original derivation scaled the n-NRF such that the mean response to a heel lance in newborns at term age was 1 (Hartley et al., 2017). In our Oxford dataset of 119 infants, the magnitude of the mean heel lance response is 1.02 (95% CI: 0.90 – 1.12). The confidence interval was calculated by bootstrap resampling using the *boot* library in R (Canty & Ripley, 2021; Davison & Hinkley, 1997). The 95% CI in these 119 infants (0.90 – 1.12) demonstrates that the n-NRF measure of the mean heel lance response is equivalent to a magnitude of one in infants aged 34.0 – 43.0 weeks PMA (Table S2, Figure S2b). This sets a broader age range to test the scale equivalence of a n-NRF measurement of heel lance response to one in independent datasets. The standard deviation of heel lance response in this sample is 0.75.


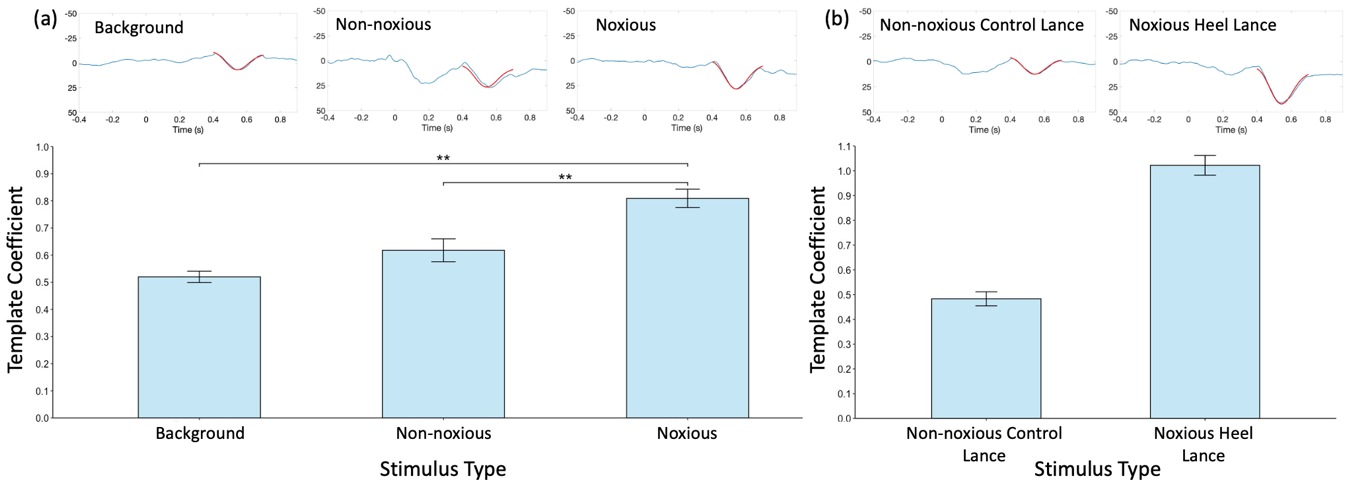


**Figure A2: (a)** Dataset (n=18) used in the original n-NRF derivation (Hartley et al., 2017). Top: Mean woody-filtered EEG traces for all participant trials for background, non-noxious, and noxious stimuli (blue), with the noxious-evoked response n-NRF fit shown (red). Bottom: Mean n-NRF coefficient over all trials for background, and non-noxious and noxious stimuli with bars showing standard error of the mean, and asterisks indicating different levels of significance between measures. Noxious response is significantly greater than non-noxious (t = 2.9, p = 0.006) and background (t = 2.8, p = 0.007) whilst non-noxious response is not significantly different from background (t = -0.9, p = 0.2) using a one-sided partially overlapping samples t-test, as described in Derrick et al. to account for data containing both paired and independent samples (Derrick et al., 2015; Derrick, Russ, et al., 2017; Derrick, Toher, et al., 2017). These comparisons match those shown by Hartley et al., 2017, with the same data, despite differences in the EEG pre-processing. The original n-NRF derivation included responses to low intensity experimental noxious stimuli and therefore, as expected, the re-analysed data had a mean response magnitude that was less than 1 (mean noxious response = 0.81; 95% CI: 0.71 – 0.93).

**(b)** Top: Mean woody-filtered EEG traces for all participant trials for control heel lance and heel lance stimuli (blue), with the n-NRF fit shown (red). Bottom: Mean n-NRF coefficient over all trials for control heel lance (n=119, mean=0.48, std=0.54) and heel lance (n=119, mean=1.02, std=0.75) stimuli with bars showing standard error of the mean. The effect size for the difference between heel lance and control heel lance response in this sample is Cohen’s d_z_=0.589.

| **Measure** | **Mean or Mean Difference (95% CI)** | **Effect Size (95% CI)** |
| --- | --- | --- |
| Heel lance | 1.02 (0.90 – 1.12) | 1.35 (1.12 – 1.56) |
| Control heel lance | 0.483 (0.41 – 0.61) | 0.90 (0.69 – 1.18) |
| Heel lance minus control heel lance | 0.539 (0.38 – 0.72) | 0.589 (0.39 – 0.77) |

**Table A2**: Summary metrics for heel lance, control heel lance, and comparisons between measures for an Oxford dataset of n=119 infants. Confidence intervals (CI) are calculated by bootstrap resampling using the *boot* library in R (Canty & Ripley, 2021; Davison & Hinkley, 1997). The effect size for the heel lance minus control heel lance is calculated as the standardised mean difference effect size for this within-participant design, i.e., the mean of the differences of the two response scores divided by the standard deviation of the differences, also known as Cohen’s d_z_ (Lakens, 2013). The effect size of the individual response scores (heel lance or control heel lance alone) is in comparison to a null response, which is thus calculated as the mean of the responses divided by the standard deviation of the responses.

**A1.3.5. Pilot Results for Hypothesis 3:**

In 2022, Schmidt Mellado and colleagues reported that the magnitude of the noxious-evoked brain activity following a heel lance increased with PMA at the time of study in infants studied longitudinally (Schmidt Mellado et al., 2022). Using an independent locally-acquired cross-sectional sample of infants (n=35, described in Section A1.1: Oxford Dataset B, and Table A1), we observed a positive correlation between infant PMA and the magnitude of the noxious-evoked brain activity following a heel lance (Pearson R = 0.51) as shown in Figure A3. This indicates that the finding reported by Schmidt Mellado and colleagues that heel lance response correlates with PMA in premature infants is observable in this cross-sectional sample and when the n-NRF is applied to the data.


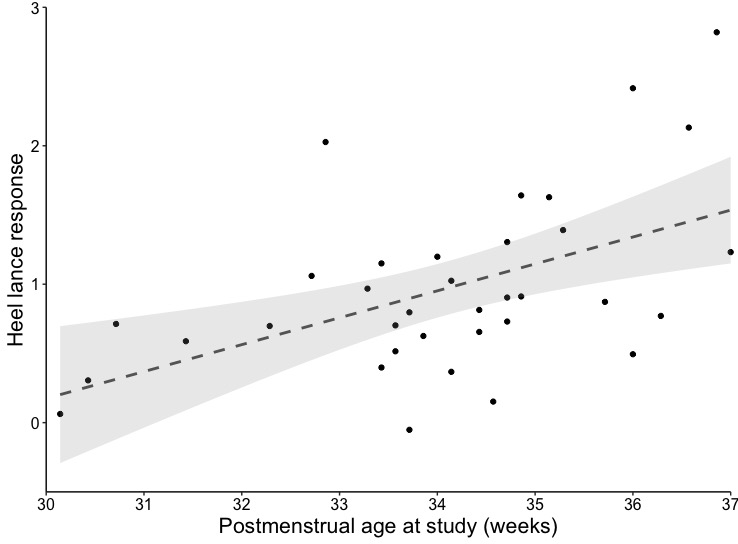


**Figure A3**: The n-NRF measure of heel lance EEG response vs PMA at time of the heel lance for the included sample of n=36 infants where each infant is represented once. The grey dashed line shows the linear least squares regression fit to the data with 95% confidence interval shaded. There is a positive correlation between PMA and heel lance response in this sample (Pearson R = 0.51, mean heel lance = 0.97, standard deviation = 0.65). The infants are aged 25.0 – 35.9 weeks GA at birth (mean = 31.1 weeks), and 30.1 – 37.0 weeks PMA at study (mean = 34.1 weeks).

**References**

Canty, A., & Ripley, B. (2021). *boot: Bootstrap Functions* (1.3-28) [Computer software]. https://CRAN.R-project.org/package=boot

Davison, A. C., & Hinkley, D. V. (1997). *Bootstrap methods and their application*. Cambridge University Press.

Derrick, B., Dobson-Mckittrick, A., Toher, D., & White, P. (2015). Test statistics for comparing two proportions with partially overlapping samples. *Journal of Applied Quantitative Methods*, *10*(3), 14.

Derrick, B., Russ, B., Toher, D., & White, P. (2017). Test statistics for the comparison of means for two samples that include both paired and independent observations. *Journal of Modern Applied Statistical Methods*, *16*(1), 137–157. https://doi.org/10.22237/jmasm/1493597280

Derrick, B., Toher, D., & White, P. (2017). How to compare the means of two samples that include paired observations and independent observations: A companion to Derrick, Russ, Toher and White (2017). *The Quantitative Methods for Psychology*, *13*(2), 120–126. https://doi.org/10.20982/tqmp.13.2.p120

Fabrizi, L., Slater, R., Worley, A., Meek, J., Boyd, S., Olhede, S., & Fitzgerald, M. (2011). A Shift in Sensory Processing that Enables the Developing Human Brain to Discriminate Touch from Pain. *Current Biology*, *21*(18), 1552–1558. https://doi.org/10.1016/j.cub.2011.08.010

Gursul, D., Goksan, S., Hartley, C., Mellado, G. S., Moultrie, F., Hoskin, A., Adams, E., Hathway, G., Walker, S., McGlone, F., & Slater, R. (2018). Stroking modulates noxious-evoked brain activity in human infants. *Current Biology*, *28*(24), R1380–R1381. https://doi.org/10.1016/j.cub.2018.11.014

Hartley, C., Duff, E. P., Green, G., Mellado, G. S., Worley, A., Rogers, R., & Slater, R. (2017). Nociceptive brain activity as a measure of analgesic efficacy in infants. *Science Translational Medicine*, *9*(388), eaah6122. https://doi.org/10.1126/scitranslmed.aah6122

Lakens, D. (2013). Calculating and reporting effect sizes to facilitate cumulative science: A practical primer for t-tests and ANOVAs. *Frontiers in Psychology*, *4*, 863. https://doi.org/10.3389/fpsyg.2013.00863

Schmidt Mellado, G., Pillay, K., Adams, E., Alarcon, A., Andritsou, F., Cobo, M. M., Evans Fry, R., Fitzgibbon, S., Moultrie, F., Baxter, L., & Slater, R. (2022). The impact of premature extrauterine exposure on infants’ stimulus-evoked brain activity across multiple sensory systems. *NeuroImage: Clinical*, *33*, 102914. https://doi.org/10.1016/j.nicl.2021.102914

Slater, R., Fabrizi, L., Worley, A., Meek, J., Boyd, S., & Fitzgerald, M. (2010). Premature infants display increased noxious-evoked neuronal activity in the brain compared to healthy age-matched term-born infants. *NeuroImage*, *52*(2), 583–589. https://doi.org/10.1016/j.neuroimage.2010.04.253
